# *Colletotrichum scovillei* and Prospective Biocontrol Agents Isolated from Asymptomatic Olive Trees

**DOI:** 10.3390/microorganisms13122838

**Published:** 2025-12-13

**Authors:** Kallimachos Nifakos, Polina C. Tsalgatidou, Athanasios Tsafouros, Christina Angeli, Epaminondas Kartsonas, Costas Delis, Ioannis Charalampopoulos, Anastasia Venieraki, Panagiotis Katinakis

**Affiliations:** 1 Laboratory of General and Agricultural Microbiology, Department of Crop Science, Agricultural University of Athens, Iera Odos 75, 11855 Athens, Greece; k.nifakos@uop.gr (K.N.); katp@aua.gr (P.K.); 2 Department of Agriculture, University of the Peloponnese, 24100 Kalamata, Greece; polina.tsalgatidou@go.uop.gr (P.C.T.); a.tsafouros@go.uop.gr (A.T.); c.angeli@go.uop.gr (C.A.); e.kartsonas@uop.gr (E.K.); k.delis@uop.gr (C.D.); 3 Laboratory of General and Agricultural Meteorology, Department of Crop Science, Agricultural University of Athens, 11855 Athens, Greece; icharalamp@aua.gr; 4 Laboratory of Plant Pathology, Department of Crop Science, Agricultural University of Athens, Iera Odos 75, 11855 Athens, Greece

**Keywords:** anthracnose, *Colletotrichum*, *Colletotrichum scovillei*, *Colletotrichum acutatum* species complex, fungal endophytes, pathogenicity

## Abstract

Olive anthracnose is a major disease worldwide; although once chiefly attributed to *Colletotrichum acutatum*, it is now clear that the predominant pathogen varies among regions. In this study, we identified *Colletotrichum scovillei* for the first time as a latent pathogen in olive fruits from groves in the Peloponnese, Greece, expanding the known diversity of *Colletotrichum* species associated with olive anthracnose. To better understand the ecological context of this finding, we examined the role of endophytic microorganisms in olive tissues and their interactions with phytopathogens. Endophytic fungi isolated from asymptomatic ripe olive fruits and leaves were characterized for phylogeny and potential pathogenicity, while competitive interactions between *Colletotrichum* species and other endophytes were assessed to identify potential biological control agents. In parallel, meteorological variability among sampling sites was analyzed to explore possible links with pathogen distribution. Our results indicate that naturally occurring endophytes sharing the *Colletotrichum* niche can suppress the necrotrophic phase of *Colletotrichum* spp., supporting the potential of such endophytes as sustainable tools for disease management. We detected *C. scovillei* in asymptomatic olives in one sampling year and confirmed its virulence via inoculation assays. This temporally limited yet virulent occurrence, alongside the activity of resident endophytes, supports an integrated, ecology-informed approach to anthracnose management.

## 1. Introduction

Numerous *Colletotrichum* species cause anthracnose across diverse hosts; currently, more than 340 accepted species are recognized, grouped into about 20 monophyletic species complexes plus several singleton lineages [1,2,3,4]. It is well known that *Colletotrichum* species exhibit different lifestyle patterns that can be broadly categorized as necrotrophic, hemibiotrophic, latent or quiescent, and endophytic, with most species possessing the ability to switch among lifestyles [5]. Interestingly, some non-pathogenic *Colletotrichum* endophytes confer protective benefits to their host plant by reducing disease incidence and damage caused by other plant pathogens [5,6,7], or improve phosphorus uptake and growth of plants such as *Arabidopsis thaliana* [8].

Olive anthracnose is among the most important fungal diseases of olive fruit worldwide and is associated with at least eight *Colletotrichum* species belonging to the *Colletotrichum acutatum* and *gloeosporioides* species complexes with *C. acutatum*, *C. nymphaeae*, and *C. theobromicola* reported as the most predominant, causing anthracnose epiphytotics in several olive-producing countries [9]. *Colletotrichum godetiae* is often prevalent in the Mediterranean but less virulent, while other species are sporadic or of uncertain pathogenicity. The recent spread of highly virulent taxa raises the risk of more severe outbreaks and complicates crop protection [4,9,10,11,12,13,14,15,16]. This taxonomic and epidemiological dynamism suggests that endophytic or quiescent *Colletotrichum* populations in asymptomatic tissues may include overlooked pathogenic species.

Climate change, such as warmer temperatures and increased humidity, has been shown to affect the establishment and severity of plant diseases. Changing temperature, humidity, and precipitation patterns can create favorable conditions for the growth and spread of pathogens as weather patterns change. These phenomena can extend the growing season for some crops, increase susceptibility to various phytopathogens, and favor the establishment of new microbe species regardless of whether they are pathogenic or living in latent form [17].

*Colletotrichum* spp. responsible for olive anthracnose has been reported to exhibit different lifestyles, ranging from latent infections at the flowering stage to a necrotrophic phase upon fruit ripening [5]. Interestingly, in anthracnose asymptomatic olive trees from different cultivars (*Olea europaea* cv. Madural), the olive fruit samples were colonized predominantly by *Colletotrichum* endophytes profoundly living in asymptomatic state [18,19]. Furthermore, co-occurrence analysis of endophytic fungi population using cultivation-dependent and independent methods, for samples collected from asymptomatic mature fruits in symptomatic olive orchards with disease incidence ranging from low to high, demonstrated that a high population of potentially pathogenic *Colletotrichum* can be maintained in an endophytic stage [20,21]. These data suggest that under certain conditions the potentially pathogenic *Colletotrichum* spp. are forced to maintain an endophytic/quiescent stage in mature olive fruits [19,22,23]. Similarly, it has been shown that different plant species susceptible to anthracnose are inhabited by abundant endophytic *Colletotrichum* spp., which, although cause potential disease, are harbored in plant tissues that remain healthy and asymptomatic [23,24,25,26,27].

Endophytic fungi live an asymptomatic lifestyle within different plant tissues (e.g., leaves, roots) and eventually establish a mutualistic relationship with their host plant [28]. The host provides nutrients and protection to the endophytes, while in return, endophytes may help improve the host’s ability to cope with both biotic and abiotic stresses, promoting plant growth. Across many fungal taxonomic lineages, closely related endophytic and phytopathogenic species occur side by side, indicating that endophytic fungi are often phylogenetically close to biotrophic and/or necrotrophic pathogens. On the other hand, fungal lifestyles do not appear to be strictly evolutionarily stable traits, with possible transitions among endophytism, latent pathogenicity, and overt pathogenicity [28]. However, there is the notion that pathogenic and endophytic lifestyles are interchangeable and may be influenced by environmental cues and chemical and/or molecular triggers produced by the host or other endophytes [29].

It is envisaging that the endophytic asymptomatic growth or the maintenance of endophytic/quiescent stage of potential pathogens may be attributed to a balanced antagonism with other cohabitating endophytes [30,31] and/or to balanced antagonism between the endophytic fungi virulence and the host plant defenses system [30,32]. In parallel, considering that temperature and humidity strongly influence the growth, sporulation, and infectivity of endophytic and pathogenic fungi-like *Colletotrichum* on olive trees, the assessment of local weather conditions during the sampling period provides useful context for understanding the epidemiology of the disease and its potential impact on management strategies [33,34].

The Koroneiki cultivar (*Olea europaea* var. *microcarpa* alba), originating from the Messinia region of the Peloponnese, represents the predominant olive variety in Greece, accounting for 50–60% of the country’s olive oil production and exhibiting exceptional adaptability to Mediterranean climatic conditions characterized by limited water availability and poor soils. Despite producing small fruits (12–15 mm diameter), Koroneiki olives achieve remarkable oil yields of 20–30%, with the resulting extra virgin olive oil demonstrating superior organoleptic properties including low acidity, intense fruity flavor profiles, and elevated concentrations of polyphenols, α-tocopherol, and squalene. The cultivar’s agronomic resilience, high productivity (50–150 kg per tree annually), and suitability for intensive cultivation systems have established it as the cornerstone of olive oil production not only in the Peloponnese but across Greece [35,36].

To date, studies on anthracnose in Koroneiki and other cultivars in Greece indicate that recent outbreaks are associated mainly with the acutatum species complex, whereas clear records of gloeosporioides-complex species on olive in Greece remain limited/unconfirmed compared with other Mediterranean countries [21,37,38]. Moreover, beyond *C. acutatum* s.l., *C. nymphaeae*, which is member of the acutatum complex, has been detected in samples from the Peloponnese region, supported by phylogenetic evidence (multilocus analysis) and pathogenicity confirmation on olive [39].

In this context, the objectives of this study were (a) to investigate whether there are some pathogenic *Colletotrichum* species in asymptomatic olive trees in the Peloponnese region, Greece; (b) to clarify the dominant *Colletotrichum* species on olive trees in the area; (c) to screen antagonistic endophytic fungi against pathogenic *Colletotrichum* species; and (d) to assess the potential influence of weather conditions during the sampling period on the establishment of specific *Colletotrichum* species in the studied areas.

## 2. Materials and Methods

### 2.1. Sampling and Isolation and Identification of Fungal Endophytes

Plant material for endophyte isolation was collected in the middle of December 2016 from symptomless mature fruits and leaves from olive orchards (*O. europaea* cultivar Koroneiki) grown in the greater Pylia area (Messinia, Greece), where olive anthracnose had been widespread in the preceding seasons. At the regional scale, quantitative ratings were not recorded in our 2016 sampling; however, contemporaneous regional surveys reported high incidence and severity associated with outbreaks in 2015–2016 [37]. Within each orchard, we purposefully sampled only asymptomatic tissues from trees/branches showing no visible anthracnose symptoms at the time of collection, despite the orchard being symptomatic overall. In total, 144 trees from 12 orchards were sampled, yielding 4320 fruits (30 fruits per tree) and 4320 leaves (30 leaves per tree) for endophyte isolation. The collected material from each orchard was homogenized by thorough mixing. From each field, one composite sample comprising 30 fruits and 30 leaves was prepared. In total, 48 composite samples were obtained. No fungicide applications were carried out prior to sampling at the sites where asymptomatic fruits were collected. Field-collected branches, from asymptomatic tissues collected from anthracnose-affected orchards at seven different locations (Koukounaria, Lachanada, Polichni, Scinolakka, Vounaria, Tapia Methoni, and Siamou; Figure S1) were transported to the laboratory in plastic containers and stored at 4 °C for no longer than 24 h prior to use. Symptomless fruits and leaves were washed thoroughly under running tap water for 20 min and surface-sterilized with 70% (*w*/*v*) ethanol for 5 min, 0.5% (*w*/*v*) NaOCl-0.2% (*w*/*v*) Tween 20 for 5 min, and 70% (*w*/*v*) ethanol for 40 s. Finally, they were rinsed off four times with sterilized distilled water [40]. The efficacy of surface sterilization was verified by plating 100 μL of the final rinse water onto potado dextrose agar (PDA, Condalab, Madrid, Spain) (amended with chloramphenicol) and incubating at 25 °C for seven days. When indicated, tissue imprints of sterilized segments were also performed as an additional control [40,41]. Small portions of the fruits, including epicarp and mesocarp tissues, were ground and placed on PDA supplemented with chloramphenicol (100 mg/L) and streptomycin sulfate (50 mg/L) and incubated at 24 ± 1 °C in the dark. Similarly, leaf fragments (6 mm^2^) from the lamina base adjacent to the petiole were excised and placed onto PDA plates. Upon observation of hyphal growth, small portions were aseptically taken, transferred into new media (PDA), and incubated at 24 ± 1 °C in the dark for seven days. Single-spore isolates were prepared to maintain pure cultures of the fungus. A total of 40 fungal isolates were collected from asymptomatic fruits and leaves. In this study, we use ‘candidate endophytes’ in the operational sense of fungi isolated from internal plant tissues following validated surface sterilization and negative contamination controls.

### 2.2. Identification of Fungal Endophytes

DNA from single-spore fungal isolates grown in PDA plates was extracted by using the Pure Link Plant Total DNA Purification Kit (Invitrogen, Thermo Fisher Scientific, Carlsbad, CA, USA) following the manufacturer’s instructions. The rDNA amplification of the ITS region was performed in a 50 μL reaction mixture, which included 2 μL DNA template (1–20 ng), 0.4 μM of each primer ITS1 (5′-TCCGTAGGTGAACCTGCGG-3′) and ITS4 (5′-TCCTCCGCTTATTGATATGC-3′) [42], 1.5 mM MgCl_2_, 0.2 μM dNTPs, 5 μL of Taq buffer, and 1.25 U Taq DNA polymerase (Qiagen, Hilden, Germany). The PCR amplification was performed on a cycler PCR machine (Bio-Rad, Hercules, CA, USA) with an initial denaturation at 95 °C for 2 min, followed by 35 cycles of amplification (95 °C for 30 s, 55 °C for 30 s, and 72 °C for 1 min) and an extension step at 72 °C for 7 min. The PCR products were purified using the QIAquick PCR Purification Kit (Qiagen) and visualized on 1.5% agarose gel. Purified amplicons were sequenced in both directions to generate consensus sequences. Consensus ITS sequences were queried using BLASTn against the NCBI nucleotide database (U.S. National Center for Biotechnology Information, Bethesda, MD, USA) to obtain preliminary similarity matches and guide the selection of reference taxa.

The isolates identified as *Colletotrichum* (based on ITS sequences) were further phylogenetically characterized using multi-gene phylogenetic analyses based on three genomic loci *beta-tubulin-2* (*TUB2*), histone (*HISTONE-H3*), and Key Lime anthracnose pathogenicity (*ΚLAP1*) [43,44,45]. These gene sequences were amplified and sequenced by using the primer pairs T1 (5′-AACATGCGTGAGATTGTAAGT-3′) and T2 (5′-TAGTGACCCTTGGCCCAGTTG-3′) for *TUB2*, CYLH3F (5′-AGGTCCACTGGTGGCAAG-3′) and CYLH3R (5′-AGCTGGATGTCCTTGGACTG-3′) for *HISTONE-H3*, and Lime_1F (5′-GCCAACAAATAAACGCCACT-3′) and Lime_2R (5′-GACTTATTCGGTGACGTGCC-3′) for *KLAP1*, as previously used by Angeli et al. [39]. PCR reactions were performed in a 2720 Thermal Cycler (Applied Biosystems, Australia) following the protocol described by Damm et al. [43]. Sequences have been deposited in GenBank (*HISTONE-H3* PP910075–PP910091; *TUB2* PP910092–PP910108; *KLAP1* OR671205, OR671206, OR853101, OR853103, OR853104, OR876268, OR876269, OR877147, OR877149, OR877150, OR877151, OR878050, PQ041292–PQ041295). A full mapping of accession numbers to isolate IDs, host/tissue, location, gene locus, and sequence length is provided in Table S1a. Because BLAST searches are not sufficient for species identification, the isolates were identified phylogenetically. Sequences from our isolates were aligned with sequences from ex-type or epitype strains of candidate species within the *acutatum* species complex and other relevant *Colletotrichum* taxa retrieved from GenBank. Alignments were generated for single loci (ITS, *TUB2*, *HISTONE-H3*, *KLAP1*) and for a concatenated multilocus dataset. Phylogenetic trees were inferred using maximum likelihood (ML) in MEGA v11, with the best-fit substitution model selected by BIC for each partition; node support was assessed with 1000 bootstrap replicates. Neighbor-joining analyses were also performed as a heuristic comparison. Species assignments were based on the topology and support values in the multilocus ML tree [46,47]. Reference species sequences were obtained from the draft sequenced genomes of reference strains of the acutatum species complex (https://www.ncbi.nlm.nih.gov/assembly/?term=Colletotrichum+acutatum+species+complex, accessed on 10 September 2025) and *Colletotrichum higginsianum* IMI 349063GCA_001672515.1 [48]. Reference sequences were selected based on their high similarity with our query sequences using BLAST against the assembly. Both the reference sequences and newly generated sequences in this study are listed in Supplementary Table S1b.

### 2.3. Virulence Testing

All *Colletotrichum* strains from the asymptomatic olive fruits were used for virulence testing. Single-spore cultures of each isolate were grown on potato dextrose agar for seven days at room temperature (25 °C) to induce conidia formation. The spores were harvested by adding 10 mL of sterilized distilled water onto the culture, which was then gently swirled to dislodge the conidia. Spore concentration was adjusted to 10^5^ conidia/mL using a hemocytometer. Olive fruits with a 2.05 maturity index value [49] were surface-disinfested as described above and dried on sterilized filter paper. Olive fruits were punctured at the equatorial zone with a sterile 16 G needle (depth of 2 mm) and inoculated with 10 μL of the conidial suspension (1 × 10^5^ conidia mL^−1^). Mock controls received sterile distilled water. Fruits were incubated at 24 ± 1 °C for nine days in a moist chamber to ensure a high relative humidity (>80%). There were two replicates (moist chambers) per treatment, with 10 fruits per replicate arranged in a completely randomized design. Lesion development was monitored daily, and Koch’s postulates were completed by re-isolating *Colletotrichum* from symptomatic tissue.

Visual assessment of symptoms (lesion area) was observed at three, six, and nine days after *Colletotrichum* inoculation (dpi). When necessary, the visible lesion area covering the fruit surface was estimated using ImageJ analysis software v.1.8.0 [50]. *Colletotrichum* spp. were successfully re-isolated from symptomatic olive fruits to fulfill Koch’s postulates. The number of infected fruits was recorded at three, six, and nine days, and disease incidence (DI) was calculated using the following formula: DI (%) number of infected fruits/ total number of fruits × 100. Fruits were assessed under a stereoscope, and each olive fruit was given a value using the 0–5 rating scale proposed by Moral and Trapero et al. [51], where 0 = no visible symptoms, 1 = visible lesion covering less than 25% of the fruit surface, 2 = 25 to 50%, 3 = 50 to 75%, 4 = 75 to 100%, and 5 = fruit completely covered with abundant conidia and mycelium on the surface. The disease severity index (DSI) was calculated using the following formula: DSI (%) = Σ[(a × b)/(N × Z)] × 100, where a is the number of infected fruits in a specific value of rating scale, b is the corresponding rating scale value, N is the total number of sampled fruits, and Z is the highest score on the rating scale [52].

### 2.4. In Vitro Evaluation of the Antagonistic Activity of Endophytic Fungal Strains Against the Selected Colletotrichum spp.

The antagonistic activity of the 22 endophytic fungal isolates obtained in this study was evaluated exclusively against *Colletotrichum* spp. using a dual-culture confrontation assay, following dos Santos Oliveira et al. 2020 [26]. The pathogen panel consisted of 17 virulent *Colletotrichum* isolates from our collection (Table 1). Briefly, six-day-old mycelia disks (6 mm diameter) of the endophytic fungi and the *Colletotrichum* isolates were inoculated at opposite poles of Petri dishes containing potato dextrose agar (PDA) culture medium, at a distance of 4 cm from each other. All endophyte–phytopathogen antagonism tests were performed in triplicate. Control cultures of both endophytic fungi and *Colletotrichum* isolates were placed on Petri dishes as single inoculants. The plates were incubated at 24 ± 1 °C for 12 days, and the antagonistic effect was assessed by measuring the area covered by the colony. Petri dishes were photographed and the area covered by the fungal pathogen in control culture and in confrontation with the endophytes was measured using the ImageJ software v.1.8.0 [50]. The percentage growth inhibition (PGI) was calculated in relation to the control using the formula: PGI = (a − b)/a × 100, where a is the colony area covered by the fungal pathogen in the control culture, and b is the area covered by the fungal pathogen in dual-culture [26].

Competitive interactions between the endophytes and pathogens were analyzed in vitro on the scale described by dos Santos Oliveira et al., 2020 [26], based on four types of interactions: A, B, C, and D. The interaction types are as follows: A = inhibition of mycelial growth with contact; B = inhibition from a distance; C = partial (CB1) and complete (CB2) endophytic growth on the pathogen after initial inhibition from a distance; D = partial (DA1) and complete (DA2) pathogen growth on the endophyte after initial inhibition with mycelial contact.

### 2.5. Effect of Colletotrichum Antagonistic Fungal Strains on Anthracnose Severity

Antagonistic endophytic fungal isolates from asymptomatic olive fruits were assayed for their antagonistic effect against *C. acutatum* KOR48l on detached olive fruits. Olive fruits were disinfected and wounded as mentioned above. Ten microliters of each endophytic fungi strain in sterile water (10^5^ cfu/mL) were applied to the wound, and the fruits were incubated for 12 h at 28 ± 1 °C. After incubation, 10 μL of a *C. acutatum* KOR48l conidial suspension (1 × 10^5^ conidia/mL) was added to the wounds. In parallel, wounded fruits used as positive controls were inoculated with 10 μL of *C. acutatum* KOR48l conidial suspension (1 × 10^5^ conidia/mL), and negative control olive fruits were inoculated with 10 μL of sterile ddH_2_O. After application of the treatments, fruits were incubated in plastic chambers (30 cm × 40 cm× 15 cm) for seven days at high relative humidity (>90%) and 28 ± 1 °C. Two plastic chambers, each containing 10 fruits, were used per treatment. High relative humidity was kept by placing wet sterile filter paper containing distilled water inside each chamber. DI% and DSI% were calculated as mentioned above.

### 2.6. Extraction of Secreted Diffusible Antifungal Compounds

The protocol used for the extraction of the agar-diffusible secondary metabolites of *Epicoccum* sp. KORD4f and *Phlebia* sp. KORD1f secreted during interaction with *C. acutatum* KOR48l was conducted as previously described by Nifakos et al., 2021 [53]. Briefly, PDA-growing medium of the confrontation lines were excised with a sterile scalpel, cut into small pieces with the addition of 2 mL sterile water, and placed in Erlenmeyer flasks containing 3.2 mL of ethyl acetate per g of agar (MilliporeSigma, Burlington, MA, USA) and 0.1% formic acid (MilliporeSigma, Burlington, MA, USA), vortexed, and sonicated in a water bath sonicator (Elmasonic S30H, Elma Schmidbauer GmbH, Singen, Germany) at room temperature for 30 min. The solution was then filtered through a Whatman filter and dried in a vacuum evaporator (Genevac HT-4, SP scientific, Ipswich, Suffolk, UK). Dried material was resuspended in methanol (Millipore Sigma, Burlington, MA, USA) and stored at −80 °C until further use.

### 2.7. Thin-Layer Chromatography and Agar Overlay Bioautography Method

Thin-layer chromatography (TLC) of the dual-culture (DC) secretome extracts were carried out essentially as described by Nifakos et al. 2021 [53]. For TLC-bioautography, developed chromatograms were overlaid with PDA medium containing 0.8% (*w*/*v*) agar and inoculated with *C. acutatum* KOR48l spore suspension (10^5^ spores/mL). The TLC plate was incubated at 25 °C for 24 h and then sprayed with yellow tetrazolium salt dye solution (MTT, 2.5 mg/mL) (Sigma-Aldrich^®^, MerkKGaA, Burlington, MA, USA), which turns purple in the presence of living cells. The TLC plates were photographed, and the Rf values of clear zones, indicating antifungal activity, were determined based on the following formula: Rf = distance traveled by the solute/distance traveled by the solvent front.

### 2.8. Atmospheric Examination of Olive Sampling Areas

In order to evaluate the atmospheric conditions over the sampling sites, we used the ERA5-Land reanalysis dataset [54], which provides hourly time resolution and a spatial resolution of 9 km. Using R language (R Core Team, 2022) [55] scripts along with the packages (libraries) Tidyverse [56] and terra [57], we analyzed the atmospheric data for the time period 2010–2020 initially on an hourly basis. The first task was to convert the raw data to tabular format in several time scales (hourly, daily, monthly, and yearly). After that, we performed statistical tests to evaluate differences between pairs of time series. Using the Bonferroni test (SIMES, 1986) [58], we concluded that the atmospheric conditions were significantly different among the four sampling sites for the selected time period. After that, we focused on potential differences in atmospheric conditions for 2016.

### 2.9. Statistical Analysis

The experiments of detached olive fruits assay were arranged as randomized design with three replications of ten fruits per strain. In order to assess the effect of fungal isolates on DSI% and DI% parameters, the data were analyzed using two-way ANOVA. Significant differences were determined by Tukey’s HSD test (α = 0.05). A cluster heatmap of fungal strains at different time points (3, 6, and 9 dpi) was constructed based on Euclidean distance using a publicly available online tool (https://bioinformatics.com.cn/plot_basic_cluster_heatmap_plot_024_en, accessed on 10 September 2025).

## 3. Results

### 3.1. Isolation and Molecular Identification of Fungi from Surface-Sterilized, Symptomless Olive Fruits and Leaves

Fungi were recovered from surface-sterilized, symptomless olive fruits and leaves collected from trees exhibiting severe anthracnose in the canopy. In total, 40 fungal isolates were obtained: 17 from leaves and 23 from fruits. Given the effective removal of epiphytes demonstrated by our sterility controls, these isolates are hereafter treated as candidate endophytes (Table 1). Identification of the isolates was based on ITS1-5.8S-ITS2 sequencing data and BLASTn analysis. As it is depicted in Table 1, the endophytic assemblage in fruits comprised taxa belonging to the genera *Colletotrichum* (10 out of 23 of isolates), *Fusarium* (8 out of 23 isolates), *Epicoccum* (2 out of 23 isolates), *Phlebia* (1 out of 23 isolates), *Alternaria* (1 out 23), and *Talaromyces* (1 out of 23). The endophytic assemblage in leaves comprised taxa belonging to the genera *Colletotrichum* (7 out 16 isolates), *Epicoccum* (4 out of 16), *Alternaria* (2 out of 16), *Fusarium* (2 out of 16), and *Nigrospora* (1out of 16) (Table 1).

### 3.2. Phylogeny of the Endophytic Colletotrichum Species and Colonial Morphology

**Figure 1:**
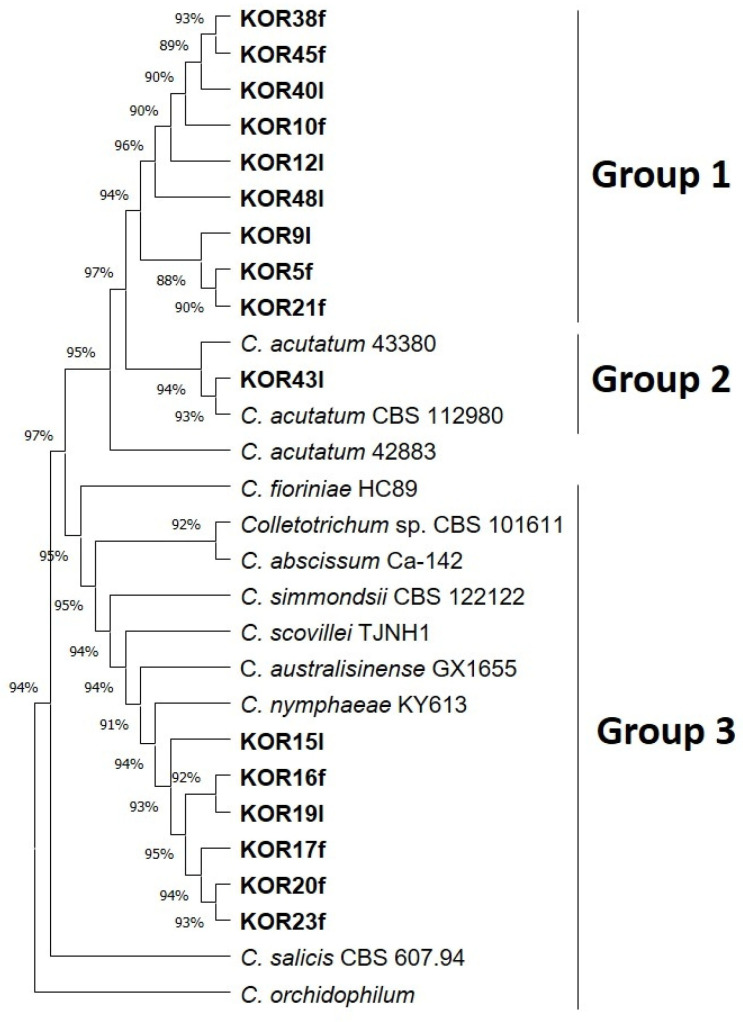
Phylogenetic tree obtained using concatenated sequences of *KLAP1*, *TUB2*, and *HISTONE-H3* genes of all isolated *Colletotrichum* strains. The strains *Colletotrichum salicis* CBS 607.94 and *Colletotrichum orchidophilum* were used as outgroup sequences. The evolutionary history was inferred by using the maximum likelihood method and Tamura–Nei model [36]. Evolutionary analyses were conducted in MEGA11 [37].

All the *Colletotrichum* isolates identified based on ITS sequences were further analyzed using sequence data from four loci: *KLAP1*, *TUB2*, and *HISTONE-H3*. The 5.8S-ITS sequences of our *Colletotrichum* isolates, together with *KLAP1*, *TUB2*, and *HISTONE-H3* datasets obtained from the draft sequenced genomes of reference strains of the acutatum species complex (Table S1b), were used either individually (for each locus) or as concatenated *KLAP1*, *TUB2*, and *HISTONE-H3* datasets for phylogenetic analysis (Figure 1 and Figure S2). The concatenated phylogenetic analysis revealed that ten *Colletotrichum* isolates (KOR38f, KOR45f, KOR40l, KOR10f, KOR12l, KOR48l, KOR9l, KOR5f, KOR21f, KOR43f) grouped together (Group 1) with reference isolates of the species *C. acutatum*, while strain KOR43f was phylogenetically separated into a different clade (Group 2) inside *C. acutatum* species complex (Figure 1). The remaining six isolates formed a well-supported clade (Group 3) that clustered with reference sequences of *C. scovillei* and *C. nymphaeae*, which is consistent with the individual phylogenetic trees of the *KLAP1*, *TUB2*, and *HISTONE-H3* genes (Figure 1 and Figure S2). These isolates (KOR16f, KOR19l, KOR17f, KOR20f, and KOR23f) are therefore assigned to *C. scovillei* and, to our knowledge, constitute a new species–host record on olive (recovered from symptomless fruits/leaves).

**Figure 2:**
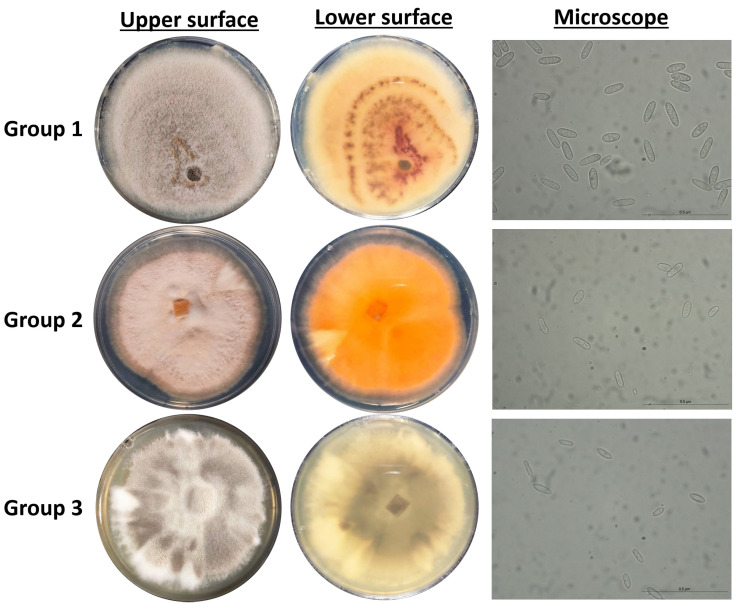
Cultural and micromorphological characters of representative *Colletotrichum* isolates, Group 1 (isolate KOR10f), Group 2 (isolate KOR43l), and Group 3 (isolate KOR19l). For each group: middle-left, obverse colony on PDA after seven days at 25 °C (5 mm inoculum); right, conidia from PDA.

Distinct morphological differences were observed between *C. acutatum* isolates belonging to Group 1 and the representative isolate KOR43 from Group 2 (Figure 2). Specifically, mycelial growth on the lower colony surface exhibited notable variation in pigmentation. Isolates from Group 1 produced a pale orange to salmon-colored mycelium on the reverse side of the colony, whereas isolate KOR43 displayed vivid orange pigmentation. Despite these differences in colony coloration, microscopic examination revealed that conidial morphology remained consistent across both Groups (Figure 2). In all cases, the conidia were characteristically elongated, indicative of the typical morphology associated with the acutatum species complex.

Isolates assigned to Group 3, identified as *C. scovillei* within the *C. nymphaeae* clade, exhibited distinct morphological traits that clearly differentiate them from the isolates of Groups 1 and 2 and from the *C. nymphaeae* species. As presented in Figure 2, the mycelium of these isolates displayed a characteristic color pattern, starting obverse white at the margin, gray to dark gray centrally, reverse whitish to slate-gray; the texture was cottony aerial at the margin and dense centrally compared with other groups.

In addition to differences in colony pigmentation and texture, conidial morphology also varied significantly (Figure 2). Conidia produced by Group 3 isolates were noticeably smaller and more ovoid in shape, hyaline, aseptate, and ellipsoidal to ovoid, contrasting with the elongated conidia observed in isolates from Groups 1 and 2. These morphological distinctions support the taxonomic separation of *C. scovillei* isolates.

### 3.3. Virulence of the Endophytic Colletotrichum on Detached Olive Fruits

**Figure 3:**
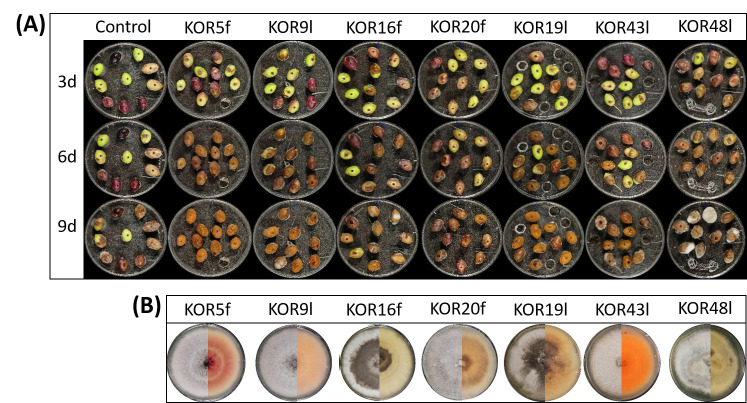
Evaluation of anthracnose symptoms after wound-mediated inoculation with *Colletotrichum* isolates. (**A**) Representative photos of anthracnose lesions on wounded olive fruits following inoculation with KOR5f, KOR9l, KOR16f, KOR20f, KOR19l, KOR43l, KOR48l, and ddH_2_0 (control) at 3, 6, and 9 dpi (d). (**B**) Representative colony photos (left: upper surface, right: lower surface) of isolates KOR5f, KOR9l, KOR16f, KOR20f, KOR19l, KOR43l, and KOR48l growing on PDA plates after 10 days of incubation at 25 °C.

The virulence of all representative *Colletotrichum* isolates was evaluated through artificial inoculation of olive fruits. All the *Colletotrichum* isolates caused anthracnose symptoms on wound-inoculated drupes of the Koroneiki olive cultivar, whereas control fruits (inoculated with sterile ddH_2_O) developed minimal or no symptoms, mostly at 9 dpi, confirming the pathogenic nature of the tested strains (Figure 3 and Figure S3). However, substantial variations in virulence were observed among the isolates.

**Figure 4:**
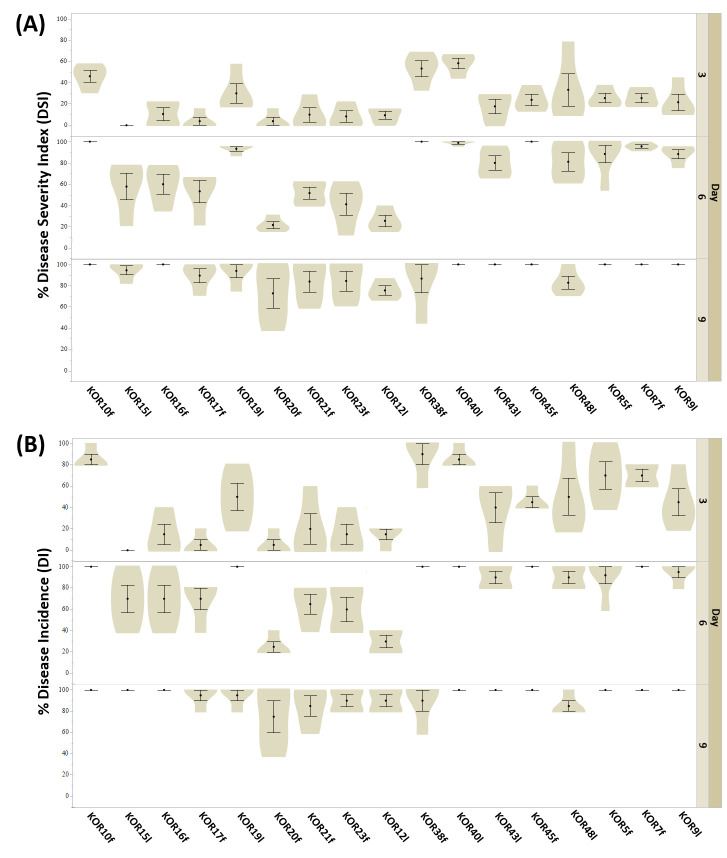
Evaluation of anthracnose symptoms on detached olive fruits after wound-mediated inoculation with *Colletotrichum* isolates at 3, 6, and 9 dpi. (**A**) disease severity index (DSI) and (**B**) disease incidence (DI) were calculated as the percentage of infected fruit in three independent experiments, each consisting of 10 olive drupes. Data in violin plots represent mean (SD) values.

The majority of the isolates, including KOR5f, KOR7f, KOR10f, KOR16f, KOR45f, KOR9l, KOR19l, KOR40l, and KOR43l, exhibited a significantly high level of virulence. These strains exhibited disease incidence (DI) values ranging from 95% to 100% and disease severity index (DSI) scores between 75% and 100% at nine days post-inoculation (dpi) (Figure 4A,B). Notably, most of these isolates began expressing visible symptoms as early as 3 dpi, indicating their high infection capability. Among them, isolates KOR10f, KOR45f, KOR38f, and KOR40l were identified as the most aggressive based on both the rapid onset and intensity of disease symptoms. Conversely, isolates KOR20f, KOR21f, and KOR23f—taxonomically assigned to *C. scovillei*—and KOR12l, classified within the *acutatum* species complex, exhibited significantly reduced virulence. These strains caused only mild symptoms, with both DI and DSI values remaining at comparatively low levels throughout the observation period (Figure 4, Table S2).

**Figure 5:**
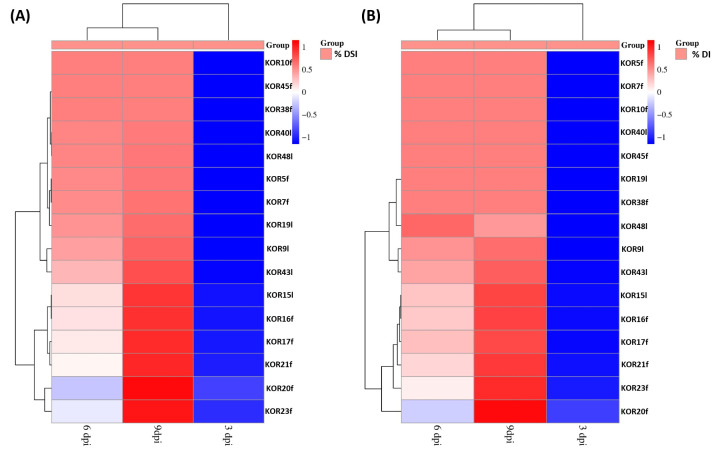
Heatmap visualization and hierarchical clustering of 16 *Colletotrichum* strains based on their virulence on detached olive fruits of Koroneiki cultivar. Clustering analysis was performed based on (**A**) disease severity index (DSI%) and (**B**) disease incidence (DI%) at 3, 6, and 9 dpi as the dependent variable to assess similarity patterns among different fungal isolates. Clustering analysis distinguished two distinct clusters, grouping the isolates into high- and low-infection ability.

Based on the heatmap described in Figure 5, the tested *Colletotrichum* isolates could be clearly categorized into two distinct groups according to their pathogenicity: fungal isolates exhibiting high-virulence and those showing low-pathogenicity. This classification was derived from the evaluation of both DSI and DI measured at three critical time points (3, 6, and 9 dpi) when inoculated on detached olive fruits. Although no significant separation between isolates was observed at 3 dpi for either DSI or DI, a clear divergence emerged from 6 dpi onwards. By 9 dpi, the differentiation between high- and low-virulence groups was consistent and statistically supported across replicates. Specifically, isolates KOR10f, KOR45f, KOR38f, KOR40l, KOR48l, KOR5f, KOR7f, KOR9l, and KOR43l belonging to the acutatum species complex, together with KOR19l belonging to *C. scovillei*, were clustered within the high-virulence group, consistently exhibiting elevated DSI and DI values over time. In contrast, isolates KOR16f, KOR17f, KOR20f, and KOR23f belonging to *C. scovillei* species, KOR15l, belonging to *C. nymphaeae* species, together with KOR21f, belonging to the acutatum species complex formed the second group, characterized by a significantly lower ability to induce disease symptoms, thus representing the low-pathogenicity category. These findings emphasize the temporal dynamics of anthracnose symptom development and highlight the importance of time-course assessments for accurate differentiation of *Colletotrichum* strain aggressiveness on olive fruit.

### 3.4. In Vitro Antagonistic Potential of Endophytic Fungi Against Colletotrichum

**Figure 6:**
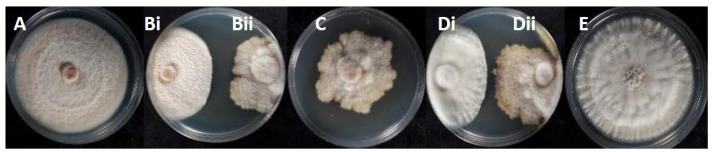
Radial growth inhibition percentages of endophytic *Colletotrichum* isolates during interaction with endophytes: (A) KOR43l; (Bi) KOR43l/(Bii) KOR42f; (C) KOR42f; (Di) KOR48l/(Dii) KOR42f; (E) KOR48l.

**Figure 7:**
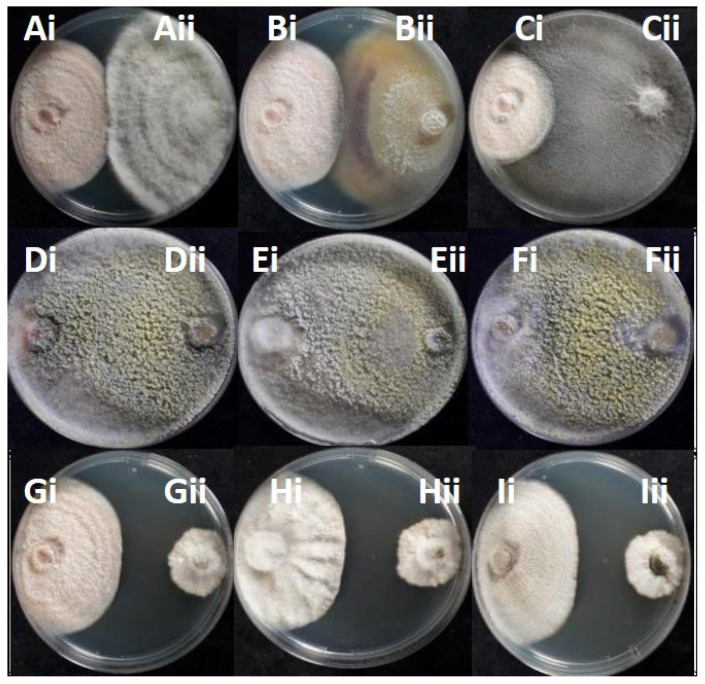
Competitive interactions of endophytic fungi with *Colletotrichum acutatum* KOR43l and KOR48l, and *Colletotrichum scovillei* KOR16f: (Ai) KOR43l/(Aii) KOR4f; (Bi) KOR43l/(Bii) KOR41f; (Ci) KOR43l/(Cii) KOR18l; (Di)KOR43l/(Dii) KOR34l; (Ei) KOR48l/(Eii) KOR34l; (Fi) KOR16f/(Fii) KOR34l; (Gi) KOR43l/(Gii) KOR11f; (Hi) KOR48l/(Hii) KOR11f; (Ii) KOR16f/(Iii) KOR11f.

All the 22 potentially antagonistic endophytic fungi cohabitating with *Colletotrichum* species were tested against the selected *Colletotrichum* strains *C. acutatum* KOR43l, KOR48l, and *C. scovillei* KOR16f, in a dual-culture competitive interaction assay. All the endophytes inhibited the radial growth of the tested *Colletotrichum* isolates, with the percentage of growth inhibition (PGI%) against pathogen *Colletotrichum* isolates ranging from 49 to 96% (Figure 6, Table S3). Most endophytes (20 out of 22 strains) present deadlock interaction with mycelial contact (competitive interaction type A) and deadlock interaction at a distance (type B). The majority of the endophytes suppressed both *C. acutatum* and *C. scovillei* strains through a consistent type of interaction. Specifically, 6 out of 22 strains (KOR41f, KOR6f, KOR39f, KOR44l, KOR18l, and KOR2l) suppressed both *Colletotrichum* species through competitive interaction type A, 9 strains (KOR11f, KOR14f, KOR8f, KORD1f, KORD4f, KOR37l, KOR33l, KOR27l, and KOR1l) through deadlock interaction type B, while type C interaction type was consistently observed only for KOR13f and KOR34l. However, a few strains exhibited different interaction types when confronted with *C. scovillei* (type A) compared to *C. acutatum* strains (type B), such as KOR46f, KOR25f, KOR4f, and KOR31 (Table S3). Endophytes KOR13f and KOR34l exhibited mycoparasitic growth against *Colletotrichum*, with both endophytes overgrowing the phytopathogen (interaction C, subtype CA1) (Figure 7, Table S3).

### 3.5. In Planta Antagonism of Endophytic Fungi Against Pathogenic Colletotrichum

**Figure 8:**
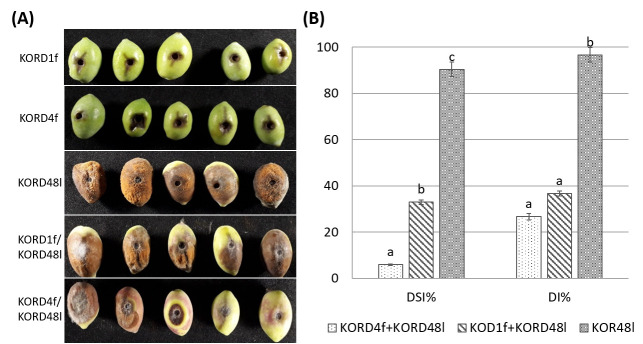
Efficacy of endophytes KORD1f and KORD4f in the reduction in anthracnose symptoms in olive fruits inoculated with *Colletotrichum acutatum* KOR48l. (**A**) Representative photos of anthracnose lesions on wounded olive drupes pre-treated with KORD1f and KORD4f prior to *C. acutatum* KOR48l infection. Olive drupes inoculated only with KORD1f and KORD4f were used as negative controls and inoculated with KORD48l as a positive control. (**B**) Disease incidence (DI%) and disease severity index (DSI%) of anthracnose symptons were calculated as the percentage of infected fruit in three independent experiments, each consisting of 10 olive drupes. Data represent mean (SD) values and letters indicate statistically significant differences after Tukey analysis.

To test the ex vivo antagonistic activity of endophytic fungi against cohabitating potentially pathogenic *Colletotrichum*, the antagonistic activity of KORD4f and KORD1f isolates against *C. acutatum* KOR48l was examined by using a detached olive fruit assay. First, we conducted experiments to infer whether inoculation with KORD4f could induce adverse effects (symptoms of anthracnose) in wounded olive fruits. When wounded olive fruits were inoculated with KORD4f or KORD1f strains, negligible visible growth of fungi and no anthracnose symptoms were observed (Figure 8A). When olive fruits were singly inoculated with conidial suspensions of *C. acutatum* KOR48l, full anthracnose symptoms were developed within seven days of incubation (Figure 8A). However, when olive fruits were inoculated with a conidial suspension of KORD4f and KORD1f 12h prior to *C. acutatum* KOR48l infection, anthracnose symptoms were significantly decreased (Figure 8A). As shown in the diagram of Figure 8B, disease incidence was significantly reduced in the presence of both KORD4f and KORD1f, with values of 27% and 36%, respectively. Similarly, the disease severity index was also significantly decreased by 6% and 33% in the presence of KORD4f and KORD1f, respectively (Figure 8B).

### 3.6. Metabolites Secreted During the Interaction Endophytic Fungi and Colletotrichum Pathogens Suppress the Growth of Fungal Pathogens

**Figure 9:**
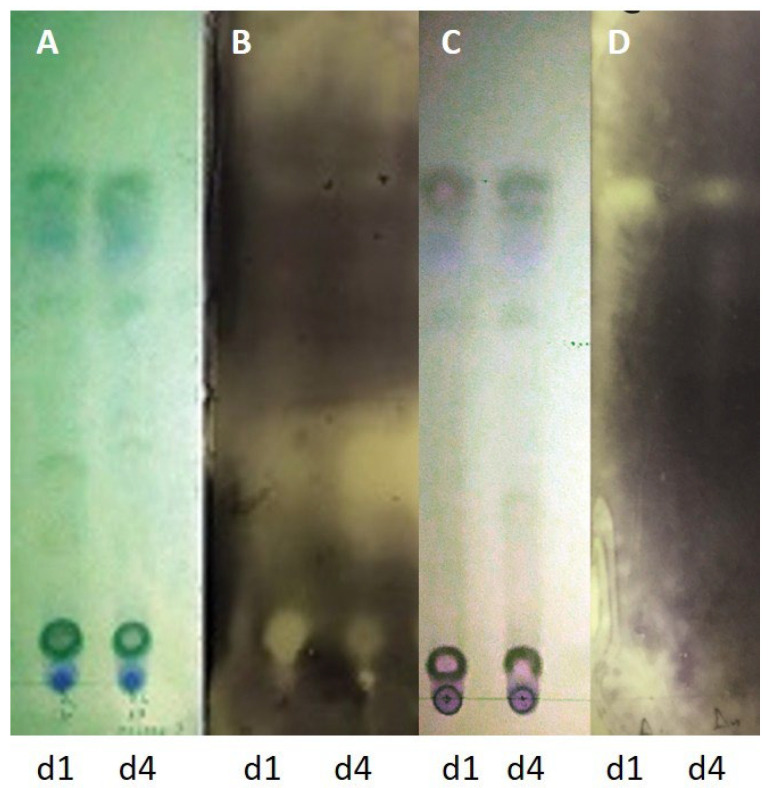
Antifungal components in the secretome of endophytic fungi after TLC-bioautography. Thin-layer chromatography plate showing the separation of ethyl acetate extracts of agar-diffusible metabolites produced during the interaction of endophytic fungi KORD1f (d1) and KORD4f (d4) with *Colletotrichum acutatum* KOR48l. TLC plate visualized under UV254 (**A**,**C**). Bioautograms using *Colletotrichum acutatum* KOR48l (**B**). Bioautography using *Fusarium oxysporum* f.sp. *radicis-lycopersici* (Forl) (**D**).

Ethyl acetate extracts of agar-diffusible metabolites produced during the interaction of endophytic fungi KORD4f and KORD1f with *C. acutatum* KOR48l were evaluated for their antifungal activity using a TLC-bioautography assay (Figure 9). The results showed that distinct inhibitory zones were formed when *C. acutatum* KOR48l was used as an indicator, suggesting that KORD4f, when confronted with fungal pathogens, may biosynthesize and secrete compound(s) with antifungal activity (Figure 9).

### 3.7. Atmospheric Conditions Differentiation as a Potential Driver of Colletotrichum Species Distribution

The ERA5-Land data analysis revealed distinct differences in climatic conditions among the sampling regions, which may account for the observed variability in *Colletotrichum* species distribution. These climatic differences are further interpreted in Table S4, which compares infection/epidemiological conditions for *C. acutatum* and *C. scovillei* (olive vs. pepper plant), explicitly focusing on infection rather than in vitro growth.

Our analysis of the atmospheric conditions associated with *Colletotrichum* species differentiation shows that the mean daily temperatures in summer (June, July, and August) 2016 were slightly higher than in adjacent years (Figure S4). Using the Bonferroni test [57], we found significant differences in atmospheric conditions among the four sampling sites, and subsequent analyses focused on the potential variability of atmospheric parameters during 2016 (Tables S5–S7). Statistical analyses revealed significant differences among the sampling sites for precipitation (Table S5), air temperature (Table S6), and relative humidity (Table S7), with only the smallest difference in relative humidity (sm_RH vs. vn_RH) being less pronounced but still significant.

Moreover, the daily maximum temperature in December 2016 (Figure S5) was lower compared to the adjacent years, while the daily minimum temperature (Figure S6) reached its lowest values during this year.

When we examine cumulative precipitation for October to December (Figure S7), there is a clear deficit in 2016. December 2016 was also the driest in terms of relative humidity (Figure S8). Thus, 2016 is characterized by an atmospheric anomaly relative to the adjacent years, with a hotter summer, a relatively dry autumn–winter period, and a cooler December. Importantly, *C. scovillei* was detected only in olive fruits collected in 2016; in subsequent surveys in the same region, we did not recover *C. scovillei* but only *C. acutatum* sensu lato. Although this correlative evidence does not prove a causal relationship, the coincidence between this anomalous year and the unique detection of *C. scovillei* on olive suggests that such atmospheric conditions may transiently favor infection or symptom expression by this species on olive.

## 4. Discussion

The ‘Koroneiki’ cultivar is essential to Greece’s olive oil industry due to its high-quality oil, adaptability, and economic importance, making it a cornerstone of Greek agriculture and a symbol of the country’s olive-growing tradition; thus, any disease of ‘Koroneiki’, especially one as serious as anthracnose, is a challenge for research. One of the main aims of the present study was to evaluate the diversity of endophytic fungi from asymptomatic olive fruits and leaves from olive orchards of the cultivar ‘Koroneiki’, which exhibited severe anthracnose symptoms. Fungicide treatments were absent prior to sampling at the sites yielding asymptomatic fruits; only olive fruit fly control interventions may have occurred and were not standardized across sites. Soil characteristics and other management practices were not included in the analysis.

The phylogenetic analysis of endophytic fungi isolates revealed that the endophytic community is dominated by *Colletotrichum* spp., comprising 44.5% of the total isolated fungi. Among *Colletotrichum* species that were in latent state inside the olive fruits and leaves, 64.70% belonged to *C. acutatum*, 29.41% were identified as *C. scovillei*, and one isolate, corresponding to 5.88% of the isolates, belonged to *C. nymphaeae*.

An interesting and perhaps unexpected finding in our study was the presence of the fungal species *C. scovillei* in four of the isolates from olive fruits (KOR16f, KOR17f, KOR20f, KOR23f) and in one sample from olive leaves (KOR19l) which have been tested. In contrast, the fungus *C. nymphaeae* was not detected in any samples from olive fruits and was found in only one sample from olive leaves (KOR15l). Species identification followed the multilocus phylogenetic framework proposed for the acutatum species complex. Olive isolates KOR16f (OR146587), KOR17f (OR146602), KOR20f (OR146588), KOR21f (OR146597), and KOR23f (OR146600) consistently clustered within the *C. scovillei* clade together with ex-type and reference strains. Given the current taxonomic treatment of the complex, which emphasizes multilocus datasets over individual loci, and their agreement with the morphological characters described for *C. scovillei*, we interpret these isolates as *C. scovillei* sensu [43]. *C. scovillei* is a fungal pathogen known to cause anthracnose in various plants, including olive trees. Although *C. scovillei* shares some common features with other *Colletotrichum* species like *C. acutatum*, there is not yet enough literature about its specific symptoms in olives. *C. scovillei* is reported to cause anthracnose of pepper in the Peja-Rahovec area in the Western Balkans [11], Ohio in the United States [12], and Ontario in Canada [13], and has also been isolated from the same plant species in China [59] and several other Asian countries as well as Brazil [60]. In 2019, a type of mango anthracnose caused by *C. scovillei* was discovered [15,61], affecting both mango leaves and fruits. More recently, it has been reported from anthracnose symptoms on banana, wampi, and onion, suggesting a broader host range [60]. However, its overall host spectrum and pathological relevance for crops other than peppers remain insufficiently clarified.

We notice that all six of these morphologically differentiated *Colletotrichum* isolates cluster together in the generated concatenated phylogenetic tree (Figure 1) among the reference species used. To date, there is no report on the establishment of *C. scovillei* fungus in olive trees. This reinforces the conception that its presence in southwestern Peloponnese at the time of harvesting may be related to climate change, which can favor the establishment of a wide range of fungal species and other microorganisms [62,63]. Findings from 32 isolates collected from olive fruits of 12 different olive varieties from the same wider area of Greece revealed the presence of *C. acutatum* and *C. nymphaeae* based on genetic analysis of five loci [39].

Apart from *Colletotrichum* isolates, the remaining 55.5% of the fungal isolates belong to six genera: *Alternaria*, *Epicoccum*, *Fusarium*, *Talaromyces*, *Nigrospora*, and *Phlebia*. In a similar study of symptomless fruits (cv. Madural) collected from orchards with high anthracnose incidence also found that the fungal endophyte community was dominated by *Colletotrichum* spp., with over 35% of associated with *Colletotrichum* spp. [19]. In addition, quantitative detection of *Colletotrichum* spp. from symptomless mature olive fruits in Italian olive orchards showing severe anthracnose symptoms revealed that the fungi population was dominated by *C. godetiae* and *C. acutatum* [21]. Previous culture-dependent studies also reported that *Colletotrichum* is the most abundant species in the endophytic community in healthy açaí leaflets; *Colletotrichum* comprises 32.4% of the total isolated fungi [27], healthy *Begonia fischeri* leaves [64], healthy rubber tree leaves [65], healthy lima bean leaves [66], and healthy *Citrus* spp. leaves [67]. In the year the authors collected the asymptomatic plant samples, humidity and temperature conditions were favorable for establishment and infectivity of *Colletotrichum* species. The authors made an attempt to re-isolate this species from symptomatic and asymptomatic olives during the current growing season, but none of the isolates belonged to the *C. scovillei* species, possibly due to current unfavorable weather conditions for the pathogen (prolonged heat and lack of moisture).

Our phylogenetic analyses demonstrated that a high percentage (62.5%) of the *Colletotrichum* sp. isolates were identified as *C. acutatum*, while the other seven appear to be associated with a new host record *Colletotrichum* sp. The prevalence of the species belonging to the acutatum species complex have also been observed upon analyses of endophytic *Colletotrichum* isolates from symptomatic olive fruits in Southern Greece (Peloponnese) [37,38]. These results suggest that *C. acutatum* could remain endophytic/dormant in some mature olive fruit, despite the majority of infected olive fruits showing severe disease symptoms caused by the development and growth of fungi belonging to the same species.

According to our virulence tests, the vast majority of *Colletotrichum* isolated from asymptomatic olive fruits and leaves have the potential to cause anthracnose symptoms on olive fruits, suggesting that the necrotrophic development of endophytic/dormant *Colletotrichum* sp. may be suppressed and remain quiescent in a number of olive drupes and leaves. This is a common phenomenon in anthracnose-susceptible plants; plant organs (such as coffee berries and leaves) of different plant species often harbor relatively large populations of *Colletotrichum* sp. capable of producing disease symptoms, as evidence by their high pathogenicity on detached fruits or leaves assays, yet the organs remain healthy and *Colletotrichum* sp. are considered as endophytes [7,23,25,68]. Furthermore, endophytic fungi co-existing with virulent *Colletotrichum* sp. in anthracnose lesions in guarana leaves showed in vitro and in planta potential to suppress the growth and development of the virulent *Colletotrichum* sp. [69]. Similarly, a Paecilomyces strain, *P. maximus* NJC01, co-existing with *C. brevisporum* in anthracnose lesions of soybean pods, also showed high in vitro and in vivo antagonistic activity against *C. brevisporum* [70]. Thus, it is reasonable to assume that olive drupes may remain asymptomatic due to the presence of an endophytic fungal microbiome able to tame pathogenic *C. acutatum* and newly recorded *Colletotrichum* spp. through competition for space and nutrients, production of antifungal compounds (antibiosis), or parasitism [71].

Our study demonstrated that the vast majority of fungal endophytes co-existing with *Colletotrichum* sp. in symptomless fruit and leaves were highly antagonistic against virulent *C. acutatum* and the newly recorded *Colletotrichum* spp., highlighting the potential of *C. acutatum*-antagonistic endophytic fungi in taming the cohabitating virulent *Colletotrichum* species in vitro. Similar results were also obtained in previous studies, in which dual-culture assays of endophytic fungi with *Colletotrichum* pathogens resulted in mycelial growth inhibition and formation of inhibition zone [27,31,65].

Recent studies demonstrate that the concatenation approach in phylogeny yields more accurate trees even when the sequences have evolved with different substitution patterns [72,73]. The concatenated phylogenetic tree presented in the present study highlights a clear grouping of related *Colletotrichum* species and differentiation from less-related members. Chen et al. 2005 [45] and a recent article from Angeli et al., 2024 [39] indicate that *KLAP1*, a putative uncharacterized conserved transcription activator, is an important pathogenicity factor in *C. acutatum*. For their experiments, they created non-pathogenic mutants and subsequently identified a fungal gene required for fungal pathogenicity and symptom development on leaves.

We noticed that species differentiation, although evident in the phylogeny of all genes tested, became sharper with the use of the *KLAP1* gene.

The integration of meteorological parameters and analysis into our study provides critical insight into the ecological processes shaping *Colletotrichum* diversity among the sampling areas. Regional differences in temperature, precipitation, and humidity likely acted as selective forces, promoting the establishment of distinct fungal taxa in each olive-growing area. These findings underscore the importance of incorporating climatic data into studies of plant–pathogen interactions, particularly when aiming to explain patterns of species differentiation and distribution.

Of the investigated endophytic fungi, *Epicoccum* and *Phlebia* were able to suppress the growth of *C. acutatum* both through competition for space and nutrients (due to their fast growth rate) and through the secretion of secondary metabolites, which impaired growth of *C. acutatum*. *Epicocum* spp. secretion products have shown the presence of antifungal compounds exhibiting strong inhibition of conidia germination in various pathogens. Similar results were also obtained in previous studies, indicating that exposure to culture extracts of *Epicoccum* resulted in inhibition of *C. gloesporiodes* and *Fusarium graminearum* conidia development [74,75].

Advanced diagnostic tools for *C. acutatum* sensu lato and *C. nymphaeae* are needed to enable rapid and accurate diagnosis of olive anthracnose, as already suggested by recent developments in species-specific molecular and real-time PCR assays for *Colletotrichum* spp. in olive and other hosts [44,76,77].

Our findings indicate that 2016 was characterized by an atmospheric anomaly compared to adjacent years, with slightly higher summer mean daily temperatures, a relatively dry autumn–winter period, and notably cooler conditions in December. Such deviations in temperature, precipitation, and relative humidity may have created a microclimate favorable for the occurrence of *C. scovillei*. Climatic extremes and anomalies are increasingly recognized as pivotal drivers in fungal disease epidemiology and shifts in pathogen distribution under global change scenarios [78]. Moreover, experimental studies have long demonstrated that temperature and humidity strongly modulate *Colletotrichum* conidial germination, appressorium formation, and infection success [79]. In the context of olive anthracnose, integrated approaches have also stressed the need to account for climatic variability when developing management models [69]. In our case, *C. scovillei* was isolated exclusively from olive fruits collected in 2016 and not in later years, which is consistent with a sporadic and possibly opportunistic occurrence on olive rather than a stable pathogen–host association. Thus, the unusual weather patterns of 2016 could plausibly help to explain the emergence or persistence of *C. scovillei* in our sampling year. The observed significant variability in precipitation, temperature, and relative humidity across the sampling sites highlights the heterogeneity of local microclimatic conditions. Such differences are likely to influence the epidemiology of *Colletotrichum* spp., as fluctuations in moisture and temperature regimes are known to modulate pathogen survival, sporulation, and infection efficiency.

## 5. Conclusions

Our results suggest that naturally occurring endophytes sharing the same ecological niche with endophytic potentially virulent *Colletotrichum* sp. in healthy-looking olive fruits and leaves from olive trees with high anthracnose incidence are endowed with the ability to suppress the necrotrophic development of endophytic/dormant *Colletotrichum* species pathogens in olive trees. Also, the presence of *C. scovillei* was confirmed in olive samples from the Peloponnese, and in vitro pathogenicity tests indicated that this species exhibits a virulence potential equivalent to that of other *Colletotrichum* taxa previously documented in Greece. Furthermore, our data revealed that besides *C. acutatum*, newly recorded *Colletotrichum* species may also cause anthracnose symptoms in olives.

## Figures and Tables

**Table 1:** Preliminary ITS (ITS1–ITS4) BLAST results for fungi recovered from surface-sterilized, symptomless olive tissues.

Isolated from Olive Fruits		Isolated from Olive Leaves	
Isolate Code (GenBank Acc. No.)	Identity to the Closest Species (GenBank Acc. No.)	Isolate Code (GenBank Acc. No.)	Identity to the Closest Species (GenBank Acc. No.)
KOR5f (OR146582)	99.57% *Colletotrichum acutatum* (EU727318.1)	KOR9l (OR146583)	99.14% *C. acutatum* (FN566876.1)
KOR7f (OR146599)	100% *C. acutatum* (EF622200.1)	KOR12l (OR146592	100% *C. acutatum* (EF622201.1)
KOR10f (OR146586_	100% *C. acutatum* (EF622201.1)	KOR15l (OR146584	100% *C. nymphaeae* (KP606646.1)
KOR16f (OR146587)	100% *C. scovillei* (MF629912.1)	KOR19l (OR146585	99.63 *C. scovillei* (ON961752.1)
KOR17f (OR146602)	100% *C. scovillei* (MF629911.1)	KOR40l (OR146601	100% *C. acutatum* (EF622201.1)
KOR20f (OR146588)	100% *C. scovillei* (MF629914.1)	KOR43l (OR146594	100% *C. acutatum* (KM594097.1)
KOR21f (OR146597)	100% *C. acutatum* (ON962736.1)	K0R48l (OR146598	99.81% *C. acutatum* (JN121184.1)
KOR23f (OR146600)	99.44% *C. scovillei* (MF629906.1)	KOR44l (OR230695	100% *Alternaria zp.* (MG025872.1)
KOR38f (OR146593)	99.45% *C. acutatum* (EF622200.1)	KOR37l (OR230693	100% *Epicoccum* sp. (MT626569.1)
KOR45f (OR146596)	99.45 *C. acutatum* (MF629920.1)	KOR34l (OR230694	100% *Fusarium* sp. (KJ598867.1)
KPO46f (OR230682)	99.62% *Fusarium* sp.(MW016692.1)	KOR33l (OR230688)	100% *Fusarium* sp. (MF919404.1)
KOR42f ( OR230687)	100% *Fusarium* sp. (MT090005.1)	KOR27l (PQ035222)	98.89% *Epicoccum* sp. (KR912314.1)
KOR41f (OR230680)	100% *Fusarium* sp. (MT134967.1)	KOR18l (OR230697)	100% *Nigrospora* sp. (KP900301.1)
KOR25f (OR230686)	100% *Fusarium* sp (MK508845.1)	KOR3l (PQ035132)	100% *Epicoccum* sp. (KP749197.1)
KOR11f (OR230684)	98.79% *Fusarium* sp. (MT558582.1)	KOR2l (PQ035131)	100% *Alternaria* sp. (MH487275.1)
KOR6f (OR230681)	99.63% *Altenaria* sp. (MW009021.1)	KOR1l (PQ035130)	100% *Epicoccum* sp. (KR912314.1)
KOR39f (OR230685)	100% *Fusarium* sp. (MK102639.1)		
KOR14f (OR2306830	100% *Fusarium* sp. (MK248478.1)		
KOR13f (OR230691)	100% *Talaromyces* sp. (KJ775705.1)		
KOR4f (OR230696)	98.37% *Fusarium* sp. (MT567303.1)		
KOR8f (OR230692)	93.57% *Epicoccum* sp. (MT355641.1)		
KORD1f (OR230690)	99.66% *Phlebia* sp. (MT458525.1)		
KORD4f (OR230689)	92.91% *Epicoccum* sp. (LC543646)		

## Data Availability

The original contributions presented in this study are included in the article/Supplementary Material. Further inquiries can be directed to the corresponding author.

## Supplementary Materials

The following supporting information can be downloaded at: https://www.mdpi.com/article/10.3390/microorganisms13122838/s1, Figure S1: Map of the areas where sampling took place (Koukounaria, Lachanada, Polichni, Scinolakka, Vounaria, Tapia Methoni, and Siamou), located in the Peloponnese, Greece. Figure S2: Phylogenetic tree obtained using sequences of *KLAP1*, *TUB2*, and *HISTONE-H3* genes of all isolated *Colletotrichum* strains. Evolutionary history was inferred by using the maximum likelihood method and Tamura–Nei model [36]. Evolutionary analyses were conducted in MEGA11 [37]. Figure S3: Representative photos of anthracnose lesions on olive fruits treated with 10 μl of *Colletotrichum acutatum* KOR38f (right) compared to the control treatment (left) nine days upon inoculation. Figure S4: The daily minimum air temperature during summer months (June, July, and August) for the sampling areas: kl = Kalliroi, kn = Koukounara, sm = Siamo, and vn = Vounaria. Figure S5: The daily maximum air temperature during December for the sampling areas: kl = Kalliroi, kn = Koukounara, sm = Siamo, and vn = Vounaria. Figure S6: The daily minimum air temperature during December for the sampling areas: kl = Kalliroi, kn = Koukounara, sm = Siamo, and vn = Vounaria. Figure S7: The total (sum) precipitation (mm) from Oct. to December for the sampling areas: kl = Kalliroi, kn = Koukounara, sm = Siamo, and vn = Vounaria. Figure S8: Mean relative humidity (%) in December for the sampling areas: kl = Kalliroi, kn = Koukounara, sm = Siamo, and vn = Vounaria. Table S1a: Sequences generated in this study and deposited in GenBank. The table provides the GenBank accession number for each isolate, isolate ID, host/tissue, sampling location, gene locus, and sequence length (bp). Table S1b: Construction of the database involved downloading sequences from reference genomes found in NCBI. The different Colletotrichum species used in the study are as follows: *C. acutatum*, *C. lupini*, *C. salicis*, *C. simmondsii*, *C. filicis*, *C. fioriniae*, *C. nymphaeae*, *C. scovillei*, *C. abscissum*, and *C. australisinense*. Table S2: Assessment of anthracnose development on detached olive fruits following wound inoculation with *Colletotrichum* isolates at 3, 6, and 9 dpi. DSI% and DI% were determined as the percentage of symptomatic fruits across three independent biological replicates, each comprising 10 olive drupes. Different letters denote statistically significant differences according to Tukey’s HSD analysis. Table S3: Antagonistic potential of endophytic fungi against isolated Colletotrichum spp. strains. PGI: Percentage of Growth Inhibition; IT: Interaction Type; A: Deadlock upon Contact; B: Deadlock at Distance; C: Overgrowth of *Colletotrichum* by the Endophyte. Table S4: Comparative conditions favoring infection by *Colletotrichum acutatum* (olive) and *Colletotrichum scovillei* (pepper/capsicum). The table summarizes conditions that favor infection/epidemiology (not necessarily in vitro growth). Table S5: Pairwise Bonferroni test results: kl_pr = Kalliroi precipitation, kn_pr = Koukounara precipitation, sm_pr = Siamo precipitation, and vn_pr = Vounaria precipitation. Table S6: Pairwise Bonferroni test results: kl_T = Kalliroi temperature, kn_T = Koukounara temperature, sm_T = Siamo temperature, and vn_T = Vounaria temperature. Table S7: Pairwise Bonferroni test results: kl_RH = Kalliroi relative humidity, kn_RH = Koukounara relative humidity, sm_RH = Siamo relative humidity, and vn_RH = Vounaria relative humidity.
