# Supplementary material for: Colletotrichum scovillei and Prospective Biocontrol Agents Isolated from Asymptomatic Olive Trees
Source: Microorganisms. 2025 Dec 13;13(12):2838. doi: 10.3390/microorganisms13122838 (PMC12735693; doi:10.3390/microorganisms13122838)
Supplement: Supplementary file 1 [file microorganisms-13-02838-s001.zip › microorganisms-3941117-supplementary.pdf]

Article

Colletotrichum scovillei and prospective biocontrol agents isolated from asymptomatic olive trees

Kallimachos Nifakos [1,2], Polina C. Tsalgatidou[2], Athanasios Tsafouros[2], Christina Angeli[2], Epaminondas Kartsonas[2], Costas Delis[2], Ioannis Charalampopoulos[3], Anastasia Venieraki [4*] and Panagiotis Katinakis[1]

[1] Laboratory of General and Agricultural Microbiology, Crop Science Department, Agricultural University of Athens, Iera Odos 75, 11855 Athens, Greece
[2] Department of Agriculture, University of the Peloponnese, 24100 Kalamata, Greece.
[3] Laboratory of General and Agricultural Meteorology, Department of Crop Science, Agricultural University of Athens, 11855 Athens, Greece
[4] Laboratory of Plant Pathology, Crop Science Department, Agricultural University of Athens, Iera Odos 75, 11855 Athens, Greece.
*Correspondence: venieraki@aua.gr

Supplementary materials

Figure S1. Map of the areas where sampling took place (Koukounaria, Lachanada, Polichni, Scinolakka, Vounaria, Tapia Methoni, and Siamou), located in the Peloponnese, Greece.

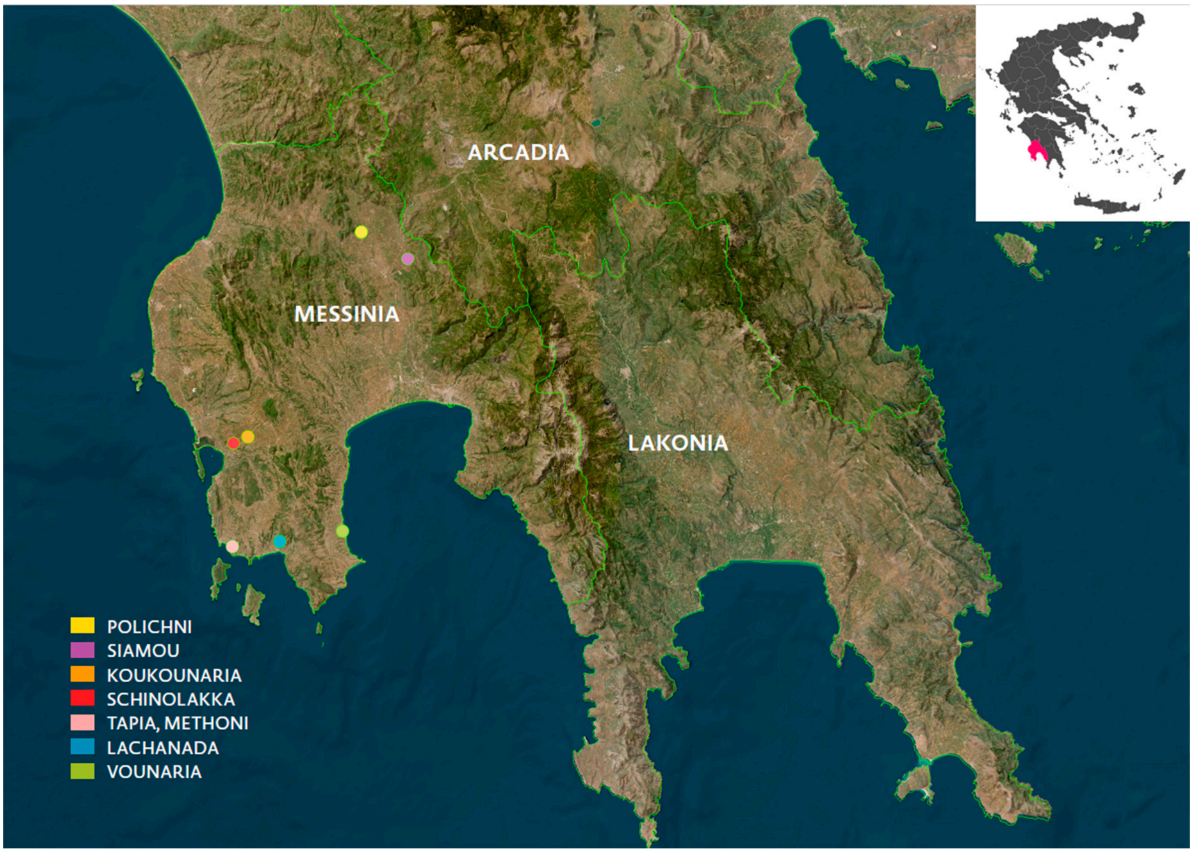

Table S1a. Sequences generated in this study and deposited in GenBank. The table provides the GenBank accession number for each isolate, isolate ID, host/tissue, sampling location, gene locus, and sequence length (bp).

Isolate ID	Host / Tissue	Location	Gene	GenBank accession	Sequence length (bp)

K0R48l	olive, leaf base	Koukounaria, Messinia	HIS3	PP910075	379
KOR5f	olive, fruit	Lachanada, Messinia	HIS3	PP910076	379
KOR7f	olive, fruit	Tapia, Methoni	HIS3	PP910077	379
KOR9l	olive, leaf	Polichni, Messinia	HIS3	PP910078	379
KOR10f	olive, fruit	Lachanada, Messinia	HIS3	PP910079	379
KOR12l	olive, leaf	Polichni, Messinia	HIS3	PP910080	352
KOR15l	olive, leaf	Polichni, Messinia	HIS3	PP910081	379
KOR16f	olive, fruit	Vounaria, Messinia	HIS3	PP910082	379
KOR17f	olive, fruit	Vounaria, Messinia	HIS3	PP910083	379
KOR19l	olive, leaf	Vounaria, Messinia	HIS3	PP910084	379
KOR20f	olive, fruit	Vounaria Messinia	HIS3	PP910085	379
KOR21f	olive, fruit	Siamou Messinia	HIS3	PP910086	379
KOR23f	olive, fruit	Koukounaria, Messinia	HIS3	PP910087	379
KOR38f	olive, fruit	Tapia, Methoni	HIS3	PP910088	379
KOR40l	olive, leaf	Schinolakka Messinia	HIS3	PP910089	379
KOR43l	olive, leaf	Tapia, Methoni	HIS3	PP910090	379
KOR45f	olive, fruit	Schinolakka Messinia	HIS3	PP910091	379
KOR48l	olive, leaf	Koukounaria Messinia	TUB2	PP910092	738
KOR5f	olive, fruit	Lachanada Messinia	TUB2	PP910093	703
KOR7f	olive, fruit	Tapia, Methoni	TUB2	PP910094	707
KOR9l	olive, leaf	Polichni, Messinia	TUB2	PP910095	678
KOR10f	olive, fruit	Lachanada Messinia	TUB2	PP910096	705
KOR12l	olive, leaf	Polichni, Messinia	TUB2	PP910097	708
KOR15l	olive, leaf	Polichni, Messinia	TUB2	PP910098	576
KOR16f	olive, fruit	Vounaria Messinia	TUB2	PP910099	702
KOR17f	olive, fruit	Vounaria Messinia	TUB2	PP910100	735
KOR19l	olive, leaf	Vounaria Messinia	TUB2	PP910101	705
KOR20f	olive, fruit	Vounaria Messinia	TUB2	PP910102	743
KOR21f	olive, fruit	Siamou Messinia	TUB2	PP910103	702
KOR23f	olive, fruit	Koukounaria Messinia	TUB2	PP910104	740
KOR38f	olive, fruit	Tapia, Methoni	TUB2	PP910105	698
KOR40l	olive, leaf	Schinolakka Messinia	TUB2	PP910106	661
KOR43l	olive, leaf	Tapia, Methoni	TUB2	PP910107	756
KOR45f	olive, fruit	Schinolakka Messinia	TUB2	PP910108	739
KOR5f	olive, fruit	Lachanada Messinia	KLAP1	OR671205	368
KOR7f	olive, fruit	Tapia, Methoni	KLAP1	OR671206	368
KOR45f	olive, fruit	Schinolakka Messinia	KLAP1	OR853101	390
KOR9l	olive, leaf	Polichni, Messinia	KLAP1	OR853103	367
KOR43l	olive, leaf	Tapia, Methoni	KLAP1	OR853104	388
KOR40l	olive, leaf	Schinolakka Messinia	KLAP1	OR876268	390
KOR38f	olive, fruit	Tapia, Methoni	KLAP1	OR876269	389
KOR23f	olive, fruit	Koukounaria Messinia	KLAP1	OR877147	369

KOR20f	olive, fruit	Vounaria Messinia	KLAP1	OR877149	370
KOR19l	olive, leaf	Vounaria Messinia	KLAP1	OR877150	379
KOR17f	olive, fruit	Vounaria Messinia	KLAP1	OR877151	368
KOR21f	olive, fruit	Siamou Messinia	KLAP1	OR878050	387
KOR15l	olive, leaf	Polichni, Messinia	KLAP1	PQ041292	388
KOR16f	olive, fruit	Vounaria Messinia	KLAP1	PQ041293	381
KOR48l	olive, leaf	Koukounaria Messinia	KLAP1	PQ041294	390
KOR10f	olive, fruit	Lachanada Messinia	KLAP1	PQ041295	367

Table S1b. Construction of the database involved downloading sequences from reference genomes found in NCBI. The different Colletotrichum species used in the study are as follows: *C. acutatum, C. lupini, C. salicis, C. simmondsii , C. filicis, C. fioriniae, C. nymphaeae, C. scovillei, C. abscissum,* and *C. australisinense.*

**Klap1**	**β-tub-2**	**HIS3**
C. lupini IMI504893	*C. lupini* IMI504893	*C. lupini* IMI 504893
C. acutatum 43380	*C. acutatum* 43380	*C. acutatum* 43380
C. acutatum 43176	*C. acutatum* 43176 UWS166	*C. acutatum* 43176
C. acutatum 42883	*C. acutatum* 42883 UWS147	C. acutatum 42883
C. salicis CBS 607.94	*C.salicis* CBS 607.94	*C. salicis* strain CBS607.94
C. simmondsii CBS 122122	*C. simmondsii* 122122	*C. simmondsii* 122122
C. filicis CBS101611	C.filicis CBS 101611	*C. filicis* CBS 101611
C. fioriniae HC89	*C.fioriniae* HC89	*C. fioriniae* HC89
C. nymphaeae KY613	*C. nymphaeae* KY613	*C. nymphaeae* KY613
C. scovillei TJNH1	*C. scovillei* TJNH1	*C. scovillei* TJNH1
C. abscissum Ca-142	*C. abscissum* Ca-142	*C. abscissum* Ca142
C. australisinense GX1655	*C. australisinense* GX1655	*C. australisinense* GX1655
C. higginsianum IMI 349063 GCA_001672515.1	*C. higginsianum* IMI 349063 GCA_001672515.1	*C.higginsianum* IMI 349063 GCA_001672515.1

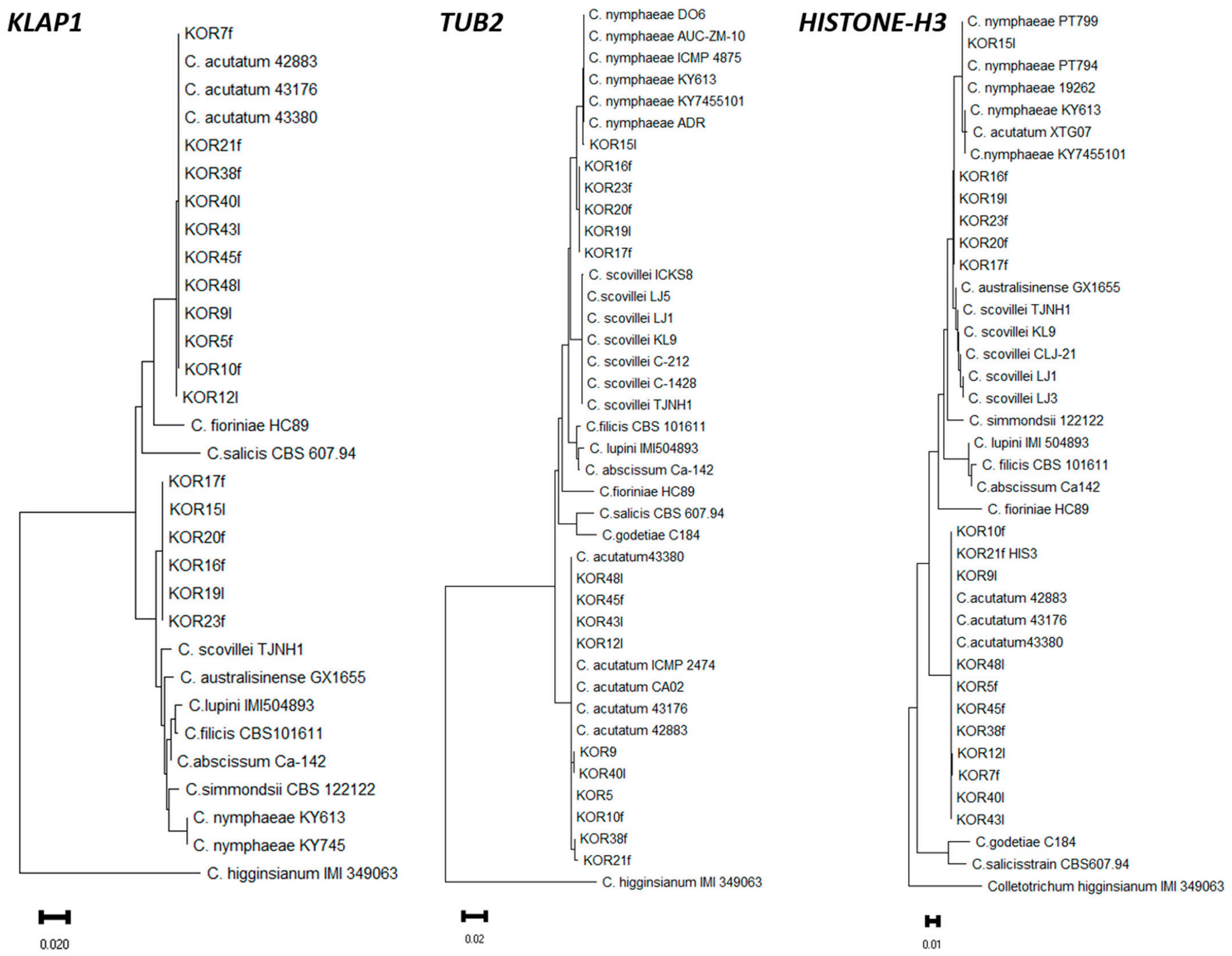

Figure S2. Phylogenetic tree obtained using sequences of *KLAP1, TUB2, and HISTONE-H3* genes of all isolated *Colletotrichum* strains. Evolutionary history was inferred by using the maximum likelihood method and Tamura–Nei model [36]. Evolutionary analyses were conducted in MEGA11 [37].

Table S2. Assessment of anthracnose development on detached olive fruits following wound inoculation with *Colletotrichum* isolates at 3, 6, and 9 dpi. DSI% and DI% were determined as the percentage of symptomatic fruits across three independent biological replicates, each comprising 10 olive drupes. Different letters denote statistically significant differences according to Tukey's HSD analysis.

Fungal strains	DI %	DSI %
KOR40l	95,00a	85,74a
KOR10f	95,00a	82,04ab
KOR38f	93,33a	80,01ab
KOR45f	90,00ab	74,63ab
KOR7f	86,67abc	73,71ab
KOR19l	81,67abc	72,41ab

KOR5f	81,67abc	70,37ab
KOR9l	80,00abc	70,00ab
KOR43l	76,67abcd	65,93abc
KOR48l	75,00abcd	65,74abc
KOR16f	61,67abcd	56,85abc
KOR15l	56,67abcde	50,74abc
KOR17f	56,67abcde	48,89abc
KOR21f	56,67abcde	48,52abc
KOR23f	55,00abcde	44,63abc
KOR12l	45,00cde	36,85abc
KOR20f	35,00de	32,78bc
Control	14,55e	16,57c

Figure S3. Representative photos of anthracnose lesions on olive fruits treated with 10 μl of *Colletotrichum acutatum* KOR38f (right) compared to the control treatment (left) nine days upon inoculation.

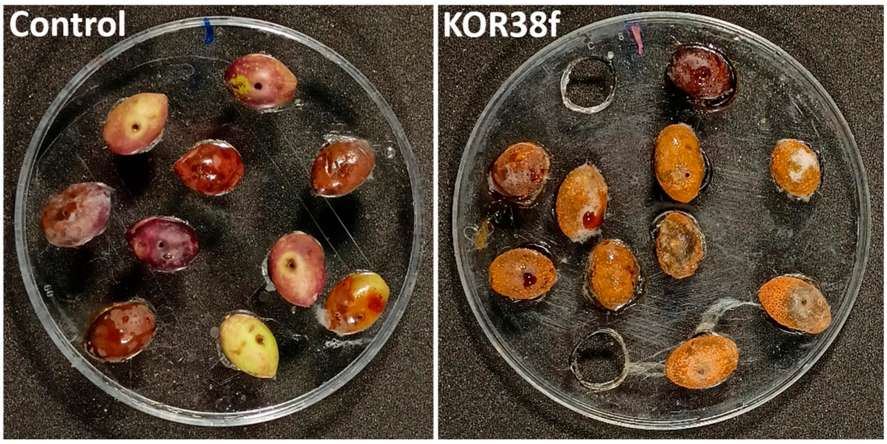

Table S3. Antagonistic potential of endophytic fungi against isolated *Colletotrichum* spp. strains. PGI: Percentage of Growth Inhibition; IT: Interaction Type; A: Deadlock upon Contact; B: Deadlock at Distance; C: Overgrowth of *Colletotrichum* by the Endophyte.

Strain	*C. scovillei* KOR16f		*C. acutatum* KOR43l		*C. acutatum* KOR48l	
	PGI (%)	IT	PGI (%)	IT	PGI (%)	IT
KOR46f	61	A	66	B	61	B
KPO42f	62	A	66	B	61	B
KOR41f	63	A	65	A	61	A
KOR25f	60	A	58	B	55	B
KOR11f	63	B	60	B	57	B
KOR6f	61	A	62	A	61	A
KOR39f	51	A	52	A	50	A
KOR14f	58	B	68	B	49	B
KOR13f	94	C	96	C	96	C

KOR4f	68	B	68	A	63	A
KOR8f	66	B	65	B	67	B
KORD1f	83	B	85	B	79	B
KORD4f	82	B	81	B	75	B
KOR44l	59	A	60	A	59	A
KOR37l	63	B	63	B	62	B
KOR34l	89	C	92	C	92	C
KOR33l	51	B	54	B	53	B
KOR27l	66	B	68	B	67	B
KOR18l	74	A	74	A	75	A
KOR3l	60	A	59	B	62	B
KOR2l	62	A	59	A	61	A
KOR1l	66	B	65	B	67	B

Table S4. Comparative conditions favoring infection by *Colletotrichum acutatum* (olive) and *Colletotrichum scovillei* (pepper/Capsicum). The table summarizes conditions that favor infection/epidemiology (not necessarily in vitro growth).

**Factor**	***Colletotrichum acutatum* (olive)**	***Colletotrichum scovillei* (pepper)**
Temperature	Optimum for infection ≈ 17–20 °C; infection occurs at 10–25 °C [1,4,8]	Severe disease around 25–28 °C; e.g., incubation/symptom expression at 27 °C [5,6,7]
Relative humidity / moisture	Free water or RH ≳ 98% required; risk increases with the duration of surface wetness [4,8,10]	High RH (≥ 90%) strongly favors disease; assays often use 27 °C and 90% RH [5,6]
Wetness period duration	Infection can occur even with short wetness at the optimum (≈ 1 h in models); severity increases steeply with longer wetness [4]	Strong disease typically requires prolonged high humidity (surface wetness / high RH chambers) [5,6]
Within-host biology	Documented latent (quiescent) infections in unripe fruits, activated at ripening [1,11]	Aggressive pathogen in warm, humid capsicum regions; quiescence is not widely reported for this host [7]

* ranges may vary by isolate and experimental setup. Values synthesize controlled inoculation and field epidemiology studies; see references [1]–[8].

Table S5. Pairwise Bonferroni test results. kl_pr= Kalliroi precipitation, kn_pr= Koukounara precipitation, sm_pr= Siamo precipitation, and vn_pr= Vounaria precipitation.

.y.	group1	group2	n1	n2	statistic	df	p	p.adj	p.adj.signif
Value	kl_pr	kn_pr	4017	4017	10.449091	4016	3.11E-25	1.87E-24	****
Value	kl_pr	sm_pr	4017	4017	2.2325078	4016	0.026	0.154	ns
Value	kl_pr	vn_pr	4017	4017	8.8871127	4016	9.29E-19	5.57E-18	****
Value	kn_pr	sm_pr	4017	4017	-9.645847	4016	8.8E-22	5.28E-21	****
Value	kn_pr	vn_pr	4017	4017	3.3108368	4016	0.000938	0.006	**
Value	sm_pr	vn_pr	4017	4017	8.6115146	4016	1.02E-17	6.12E-17	****

Interpreting your results

Significant differences (after adjustment, p.adj):

- kl_pr vs. kn_pr: **** → highly significant
- kl_pr vs. vn_pr: **** → highly significant
- kn_pr vs. sm_pr: **** → highly significant
- kn_pr vs. vn_pr: ** → significant
- sm_pr vs. vn_pr: **** → highly significant

Conclusion: All pairs have significantly different precipitation measurements.

Table S6. Pairwise Bonferroni test results. kl_T= Kalliroi temperature, kn_T= Koukounara temperature, sm_T= Siamo temperature, and vn_T= Vounaria temperature.

.y.	group1	group2	n1	n2	statistic	df	p	p.adj	p.adj.signif

Value	kl_T	kn_T	96408	96408	-284.0984	96407	0	0	****
Value	kl_T	sm_T	96408	96408	-541.7802	96407	0	0	****
Value	kl_T	vn_T	96408	96408	-277.4434	96407	0	0	****
Value	kn_T	sm_T	96408	96408	158.49372	96407	0	0	****
Value	kn_T	vn_T	96408	96408	-218.858	96407	0	0	****
Value	sm_T	vn_T	96408	96408	-200.7021	96407	0	0	****

Conclusion: All pairs have significantly different air temperature measurements.

Table S7. Pairwise Bonferroni test results. kl_RH= Kalliroi relative humidity, kn_RH= Koukounara relative humidity, sm_RH= Siamo relative humidity, and vn_RH= Vounaria relative humidity.

.y.	group1	group2	n1	n2	statistic	df	p	p.adj	p.adj.signif
Value	kl_RH	kn_RH	96408	96408	86.792987	96407	0	0	****
Value	kl_RH	sm_RH	96408	96408	149.96351	96407	0	0	****
Value	kl_RH	vn_RH	96408	96408	29.396914	96407	4.1E-189	2.5E-188	****
Value	kn_RH	sm_RH	96408	96408	-41.77488	96407	0	0	****
Value	kn_RH	vn_RH	96408	96408	-37.90466	96407	0	0	****
Value	sm_RH	vn_RH	96408	96408	3.4515679	96407	0.000558	0.003	**

Interpreting the results

1. **Highly significant differences (****)**

   - kl_RH vs. kn_RH: t = 86.8, p.adj = 0 → significant
   - kl_RH vs. sm_RH: t = 150, p.adj = 0 → significant
   - kl_RH vs. vn_RH: t = 29.4, p.adj = 2.48e-188 → significant

- o kn_RH vs. sm_RH: t = -41.8, p.adj = 0 → significant
- o kn_RH vs. vn_RH: t = -37.9, p.adj = 2.61e-311 → significant

2. **Moderately significant difference (**)**

    - o sm_RH vs. vn_RH: t = 3.45, p.adj = 0.003 → significant at 0.01 level

3. **Direction of difference**

    - o Positive t-statistic → group1 > group2 on average
    - o Negative t-statistic → group1 < group2 on average

Example interpretations:

- kl_RH is higher than kn_RH, sm_RH, and vn_RH on average.
- kn_RH is lower than kl_RH but higher than sm_RH and vn_RH.
- sm_RH and vn_RH are close, but sm_RH is slightly higher (t = 3.45).

Conclusion: There are statistically significant differences in relative humidity across almost all pairs of series. Only the smallest difference (sm_RH vs. vn_RH) is less extreme but still significant.

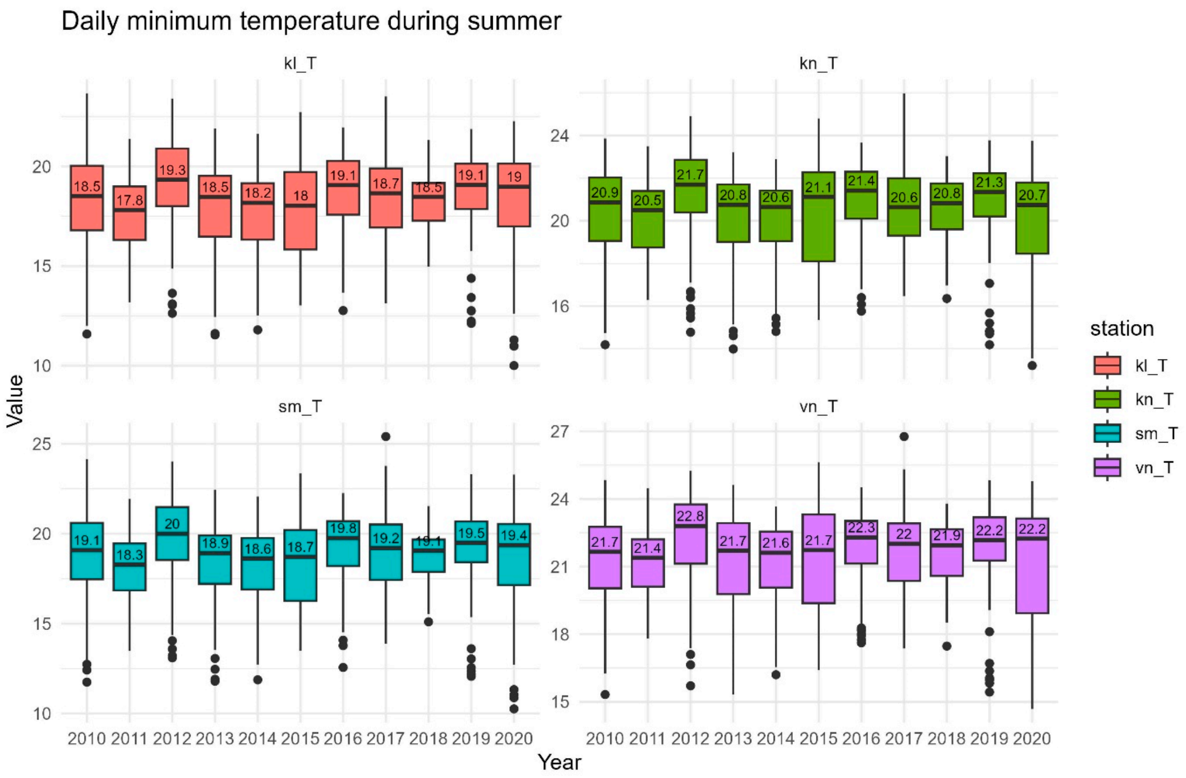

Figure S4. The daily minimum air temperature during summer months (June, July, and August) for the sampling areas: kl= Kalliroi, kn= Koukounara, sm= Siamo, and vn= Vounaria.

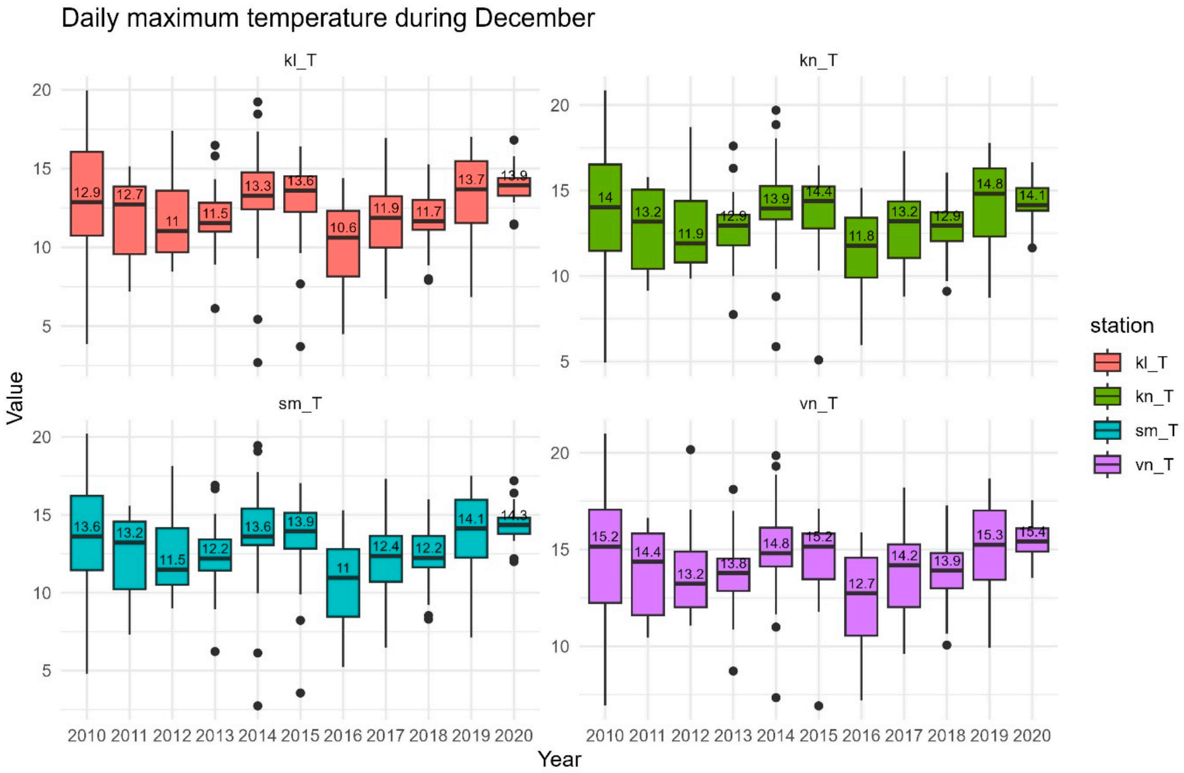

Figure S5. The daily maximum air temperature during December for the sampling areas: kl= Kalliroi, kn= Koukounara, sm= Siamo, and vn= Vounaria.

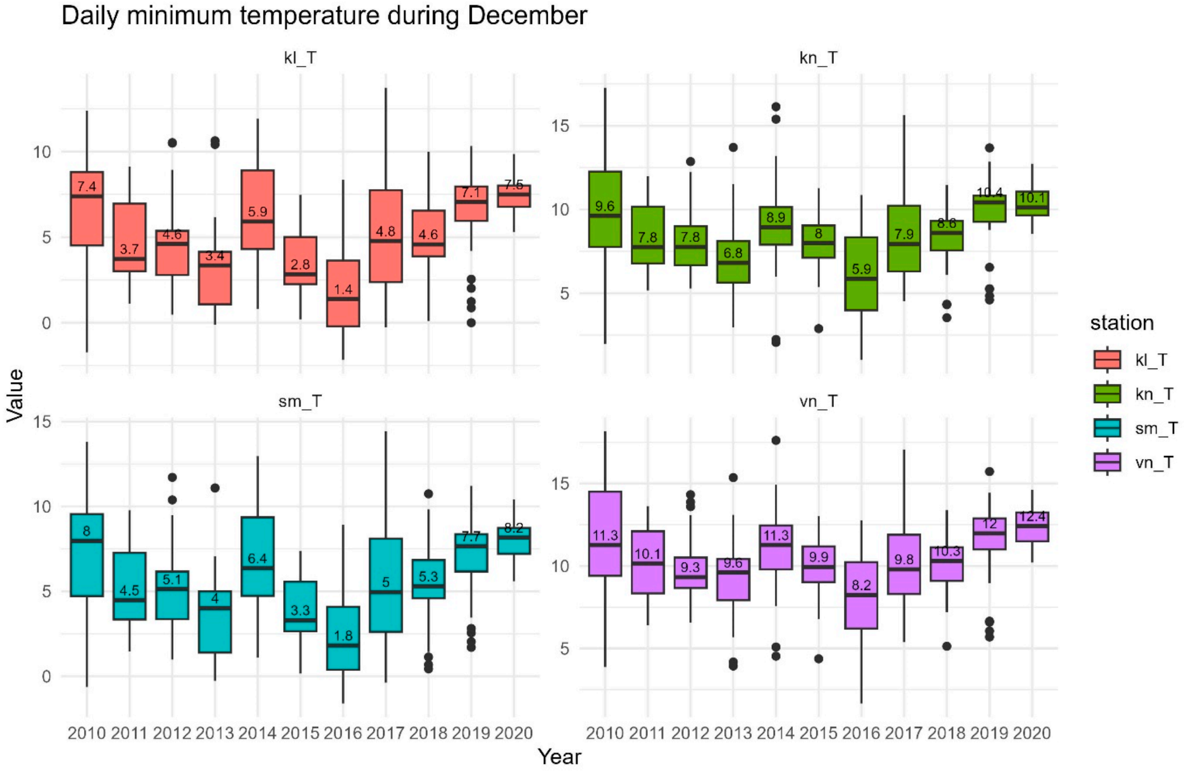

Figure S6. The daily minimum air temperature during December for the sampling areas: kl= Kalliroi, kn= Koukounara, sm= Siamo, and vn= Vounaria.

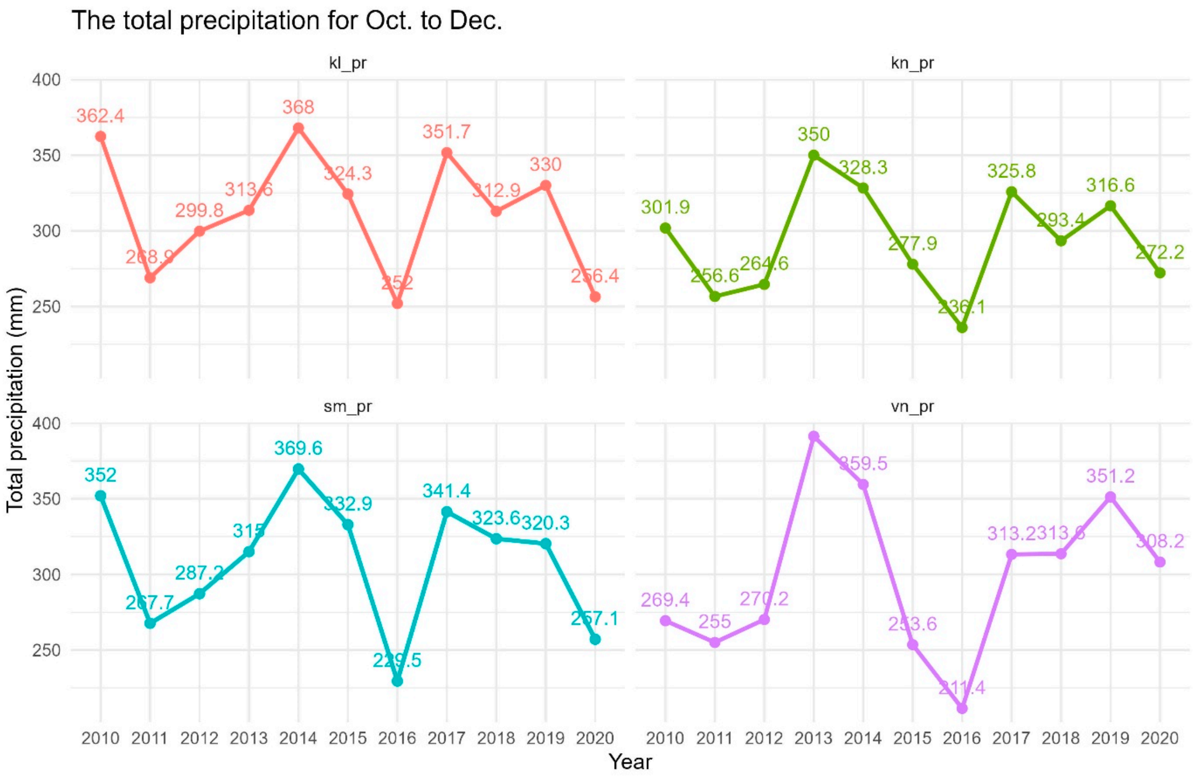

Figure S7. The total (sum) precipitation (mm) from Oct. to December for the sampling areas: kl= Kalliroi, kn= Koukounara, sm= Siamo, and vn= Vounaria.

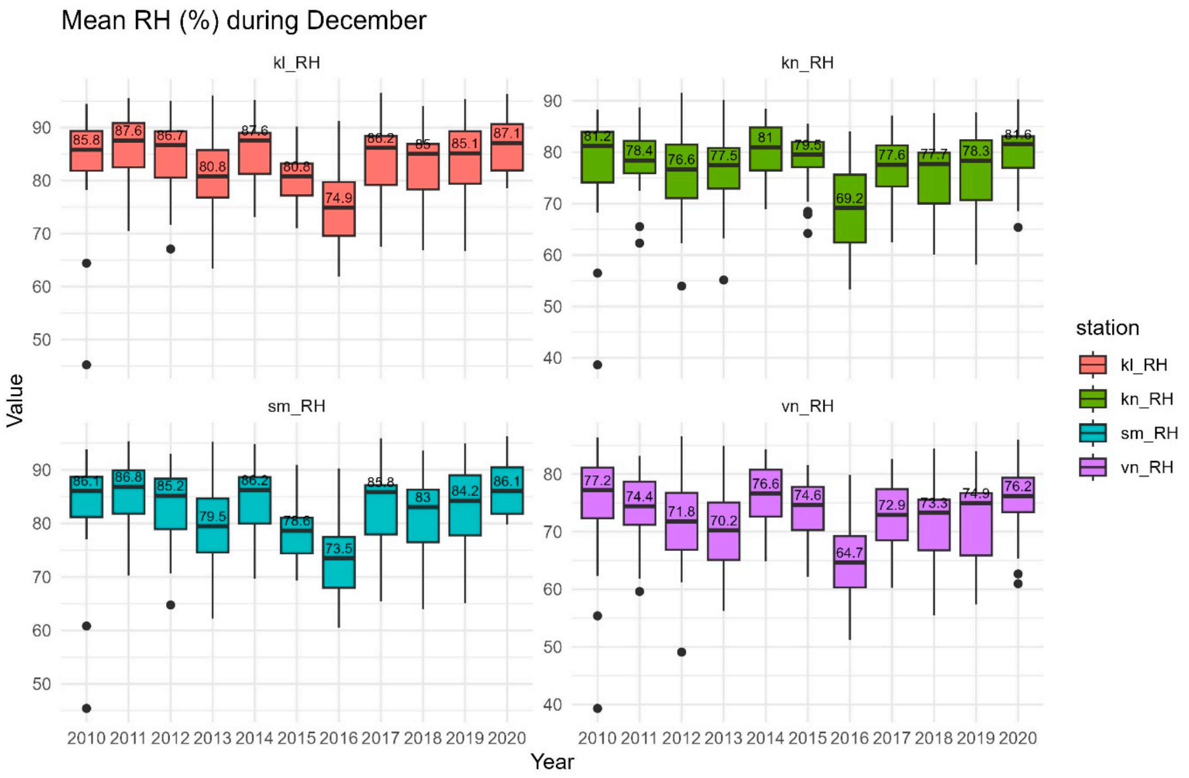

Figure S8. Mean relative humidity (%) in December for the sampling areas: kl= Kalliroi, kn= Koukounara, sm= Siamo, and vn= Vounaria.
